# Distribution and subacute modulation of endocannabinoid metabolizing enzymes in the trigeminal complex and midbrain in a pre-clinical model of post-traumatic headache

**DOI:** 10.1186/s10194-026-02356-5

**Published:** 2026-04-11

**Authors:** Gurueswar Nagarajan, Yumin Zhang

**Affiliations:** 1 Henry M. Jackson Foundation for the Advancement of Military Medicine, Bethesda, MD 20814 USA; 2 Department of Anatomy, Physiology and Genetics, Uniformed Services University of the Health Sciences, 4301 Jones Bridge Road, Bethesda, MD 20814 USA

**Keywords:** Endocannabinoid system, Hydrolyzing enzymes, Trigeminal complex, Midbrain, Mild traumatic brain injury

## Abstract

**Background:**

Post-traumatic headache (PTH) is a debilitating neurological sequela of mild traumatic brain injury (mTBI) characterized by secondary cephalic pain. The endocannabinoid system (ECS) is a critical modulator of nociception, yet the specific spatiotemporal changes in its metabolic machinery within cephalic pain circuits following mTBI are poorly understood.

**Methods:**

Using in-situ hybridization (ISH), we first characterized the levels of gene expression of major endocannabinoid (eCB) synthesizing enzymes (*Napepld*, *Gde1*, *Dagla*, *Daglb*), hydrolyzing enzymes (*Faah*, *Mgll*) and cannabinoid receptors (*Cnr1* and *Cnr2*) in the trigeminal complex (trigeminal ganglion - TG, trigeminal root entry zone - TREZ, and trigeminal nucleus caudalis - TNC) and midbrain (periaqueductal gray - PAG, dorsal raphe - DR) regions involved in the modulation of pain. Subsequently, employing a mouse model of repetitive closed head mTBI that induces cephalic pain, we assessed global eCB enzymatic gene expression changes via qPCR and region-specific changes via ISH at one-week post-injury.

**Results:**

Baseline characterization revealed complex co-expression patterns, with *Gde1* and *Daglb* transcripts being significantly more abundant than *Napepld* and *Dagla* in the TG, TNC, and PAG. Seven days post-mTBI, coinciding with the onset of PTH-like symptoms, we identified a significant upregulation of the hydrolyzing enzymes *Faah* in the peripheral TREZ but not in the TG sensory neurons. Centrally, although no difference was observed in the TNC, mTBI induced an upregulation of *Mgll* in the ventrolateral PAG (vlPAG) and the DR, particularly within DR *VGlut3*+ neurons.

**Conclusion:**

These findings demonstrate a novel neuropathological mechanism whereby mTBI triggers a persistent, region-specific upregulation of genes encoding eCB-degrading enzymes. Thus, sub-acute modulation of eCB hydrolyzing enzymes in critical peripheral and central pain-modulating regions may contribute to the maintenance of cephalic pain associated with mTBI.

**Clinical trial number:**

Not applicable.

**Supplementary Information:**

The online version contains supplementary material available at 10.1186/s10194-026-02356-5.

## Introduction

The endocannabinoids (eCBs), anandamide (N-arachidonylethanolamine - AEA) and 2-arachidonoylglycerol (2-AG), are the most extensively studied small endogenous lipid signaling molecules that are synthesized on demand. The endocannabinoid system (ECS) is composed of AEA and 2-AG carefully orchestrated by enzymes involved in their synthesis, trafficking and degradation of these lipids signaling molecules, and eventually binding to their cognate receptors [1]. The complex is found throughout the body and is involved in autocrine and paracrine function to play an important role in normal homeostasis and is implicated in a myriad of pathological conditions [2–4].

Within the ECS, eCBs can be metabolized by different complementary pathways [4–7]. In the canonical pathway, AEA is primarily synthesized by N-acyl phosphatidylethanolamine phospholipase D (Napepld) and, in part, by glycerophosphodiester phosphodiesterase 1 (Gde1). The most abundant eCB, 2-AG, is predominantly synthesized by diacylglycerol lipases (Dagla/Daglb). Subsequently, AEA is degraded by the hydrolyzing enzyme fatty acid amide hydrolase (Faah) and, in part, by N-acylethanolamine-hydrolyzing acid amidase (NAAA) [7]. Conversely, 2-AG is degraded by monoacylglycerol lipase (Mgll) and, in part, by α/β hydrolase domain-containing 6/12 (ABHD6 and ABHD12) [7, 8]. In the brain and spinal cord, the ECS is well known for its retrograde synaptic signaling [9, 10] and has long been associated with the modulation of pain [11, 12].

Cephalic pain or posttraumatic headache (PTH), induced by mild traumatic brain injury (mTBI), is a debilitating comorbid symptom that affects more than a third of the TBI clinical population [13–16]. As such, pre-clinical models have been developed to better understand its pathophysiology and translational relevance. Recently, several studies using pre-clinical models of closed-head repetitive mTBI have demonstrated the development of facial allodynia [17, 18] and spontaneous cephalic pain (grimace) long after head injury [19]. Such mTBI-induced neuropathic pain is linked to a widespread inflammatory profile [19–21] with a wide range of neural circuits involved in the development of PTH [22]. Hence, trauma induced PTH is characterized as a secondary headache disorder, distinct from primary headache disorder such as migraine and tension-type headache.

Due to a scarcity of targeted clinical trials, the treatment of PTH relies on therapies used for primary headache disorder [23]. Particularly, the current clinical management of PTH depends on symptoms severity. Acute and less frequent headaches are often managed with non-steroidal anti-inflammatory drugs. Furthermore, acute treatments such as over-the-counter analgesic and triptans have been shown to provide headache resolution in a third of the patients [24]. However, the clinical population frequently presents with persistent headaches. Preventive and prophylactic treatments such as tricyclic antidepressants, anticonvulsants, and antihypertensive medication have been shown to provide partial effectiveness against PTH [24]. The use of these medications is also frequently associated with adverse side effects including cognitive and autonomic dysfunctions. Even recently developed monoclonal antibodies against calcitonin gene-related peptide (CGRP) have proven to be only effective in 50% of the patients with PTH [25]. Thus, there is a pressing need for understanding the underlying pathophysiology and for the development of targeted treatments for PTH [23, 24].

Plant-based cannabinoids have long been used for its analgesic effect. However, given the limitations on the use of cannabinoids for the treatment of pain [26–29], the ECS has gained increasing attention as a highly promising therapeutic target [30–33]. Earlier studies have shown that brain eCB levels are elevated following severe TBI [34–36], an increase thought to contribute to the neuroprotective effects [2, 37]. However, such an increase is transient. A growing body of research has demonstrated that brain eCBs exhibit region specific modulation following open head injury (AEA [38]; 2-AG [32, 35]), closed-head injury (2-AG [36]) and headache models [39, 40]. Currently, inhibitors of FAAH and MAGL are being actively investigated in pre-clinical studies to evaluate their therapeutic effectiveness against headache-like features induced by mTBI models [18], nitroglycerine model [41, 42] and other migraine models [40, 43]. Nonetheless, because of the promiscuous nature of eCB targets and enzymatic redundancy, there is a growing rationale for developing drugs that modulate multiple targets within the endocannabinoidome [44] to effectively treat TBI pathology [2, 37]. Therefore, there is a critical need to understand the distinct expression patterns of eCB metabolizing enzymes in the pain associated neural regions.

Earlier studies have shown the broad distribution patterns of *Faah* [45], *Napepld* [46], *Dagla/*Dagl*b* [47–50], *Mgll* [51], cannabinoid type 1 (*Cnr1*) [52, 53], and type 2 (*Cnr2*) receptors [54, 55] in the brain and spinal cord. High expression of the ECS components is predominantly found in laminar distribution within cortical layers, cerebellar cortex, piriform cortex, olfactory bulb, amygdalar complex, hippocampal formation, basal ganglia, and spinal dorsal horn [56]. Consistent with this anatomical presence of the ECS, functional studies have shown dysregulation of these higher order brain regions in PTH patients [57] and migraineurs [58]. Although it is widely known that the ECS is abundant in the brain, a growing body of literature highlights its region-specific roles [32, 35, 36, 38, 59–64]. Particularly, targeting the trigeminal endocannabinoid system has been an area of increasing interest [43]. However, a comprehensive understanding of how major eCB modulating enzymes are expressed across the peripheral and central pain pathways and how they modulate mTBI-induced cephalic pain remains unclear.

Here we focused on the characterization of major eCB metabolizing enzymes in the ascending and descending pathways involved in the modulation of PTH. Specifically, we targeted the major metabolizing enzymes in the ascending pain-associated sensory regions, the trigeminal ganglion (TG) and the trigeminal nucleus caudalis (TNC), as well as the midbrain periaqueductal gray (PAG), a key region in the descending inhibitory pain pathway. Further, during the course of this study, we also found that the expression of *Mgll* extends beyond the PAG. Thus, we also included the adjoining dorsal raphe (DR) in this study. Similarly, the presence of hydrolyzing enzymes not only in TG sensory neurons but also in the nerve fibers and the trigeminal root entry zone (TREZ) prompted the inclusion of these regions. In our previous studies, we observed that mice develop cephalic pain one-week post-injury, a symptom that persists for at least a month after mTBI [18, 19]. Therefore, in this study, we examined the gene expression changes in the eCB-metabolizing enzymes at this critical one-week post-injury time point.

## Materials and methods

### In-situ hybridization and immunohistochemistry

The mRNA localization of the major eCB synthesizing and hydrolyzing enzymes in the pain-associated regions was characterized at single-molecule resolution using RNAscope Multiplex Fluorescent v2 assays (Advanced Cell Diagnostics, Inc, Newark, CA). To further identify the precise localization of mRNA signals we also combined the *in-situ* hybridization (ISH) assay with immunohistochemistry (IHC, for details see below). Following euthanasia, whole brain or TG samples were obtained as fresh-frozen or following perfusion with 4% paraformaldehyde, post-fixed for 6–8 hours, cryopreserved in 30% sucrose and immediately stored at −80 °C. Based on preliminary experiments, in situ fluorescent signals in TG samples were stronger in fresh tissue than fixed tissue. However, signals in the brain did not differ when fresh or fixed samples were used for hybridization assays. Consequently, fixed samples were preferred for the brain when a combination of ISH and IHC was used.

Tissue samples were processed for hybridization assays according to the manufacturer’s instructions. TG sections (12 μm) were processed using the fresh-frozen protocol, whereas TNC and PAG containing sections (15 μm) were on either fresh-frozen or fixed-frozen protocol. In each protocol, protease treatment was applied for 20 minutes. The RNAscope ISH probes used to identify mRNA molecules of eCB-metabolizing enzymes and their receptors are listed in Supplementary Table 1.

To further identify the expression of eCB-metabolic enzymes in specific cell types (neurons or glia), we performed IHC immediately following RNAscope Multiplex Fluorescent v2 assays as per the manufacturer’s instructions. Sections were incubated with primary antibodies for 2 hours. The following antibodies were used to identify neurons (1:2500 dilution, EnCor Biotechnology, chicken anti-NeuN, RRID:AB_2747400 or 1:100 dilution, Abcam, mouse anti-NeuN, RRID: AB_10711040) and microglia (1:250 dilution, Novus NB100-1028, goat anti-Iba-1, RRID:AB_521594). Images used for quantitative analyses were acquired using a Zeiss Axioscan Z1 slide scanner. Due to the small size of RNAscope ISH fluorescent signals, z-stack images at 0.7 µm intervals were acquired and maximum intensity projection was applied to all images.

### Repetitive traumatic brain injury

All animal procedures were conducted in accordance with NIH and AVMA guidelines and approved by the Uniformed Services University of the Health Sciences Institutional Animal Care and Use Committee (IACUC). Ten-week-old male mice (C57BL/6J; The Jackson Laboratory) were randomized into repetitive mTBI or sham groups. For the mTBI procedure, mice were anesthetized with 3% isoflurane in an induction box, subsequently placed in a supine position maintained under 1.5–2% isoflurane on a CHIMERA (Closed-Head Impact Model of Engineered Rotational Acceleration) stage. Each mouse was subjected to a 0.7 J impact directed at bregma. Sham mice underwent the same anesthesia and positioning without impact. The time to righting reflex (post-traumatic coma duration), a measure of recovery from the procedure, was recorded for all mice in both the sham and mTBI groups. Upon recovery, mice were returned to their respective home cages. This procedure was repeated once daily for 4 days, as our previous studies have shown that this repeated mild injury induces cephalic pain and allodynia by one-week post-injury [18, 19, 65]. One-week post-mTBI, tissue samples were collected for ISH, as described in the previous section.

### Behavioral assessments

The mouse grimace scale (MGS) was utilized to assess spontaneous cephalic pain responses in mTBI mice [19]. Briefly, mice were acclimated to the testing environment prior to the mTBI or sham procedure, and baseline MGS scores were measured. Since mTBI mice did not exhibit all five standard grimace features [66], scores were derived from still images based on two specific features: orbital tightening and ear position. Accordingly, MGS scores are calculated as the average of these two features. Data is expressed as the difference between average MGS scores obtained before and after the injury (5 days post-injury).

The von Frey test was used to measure peri-orbital mechanical thresholds (tactile allodynia) using a simplified Chaplan up-down method [67]. Mice were acclimated to the testing chamber twice prior to the mTBI procedure. A series of calibrated von Frey filaments (Bioseb, France) ranging from 0.02 g to 2 g were applied to the midline periorbital region where they were placed perpendicularly and pressed until the filament buckled. A positive behavioral response was identified when the mouse showed a withdrawal, shaking or retraction of the head. Each mouse was assigned a pseudo code and scoring was performed by an experimenter blinded to the treatment groups. All behavioral testing was performed between 10 AM and 3 PM.

### Semi-quantitative image analyses

Semi-quantitative image analyses were performed using QuPath [68] with the following workflow: 1) ‘Annotation’ to define area of interest; 2) ‘Cell Detection’ and ‘Positive Cell Detection’ to define individual cells; and 3) ‘Subcellular detection’ to identify and quantify transcript puncta. Because dual labeling with RNAscope (ISH) and immunohistochemistry (IHC) was unavailable during the initial phase of the study, early analyses of TG neurons in QuPath relied solely on DAPI signals. Specifically, within the complex, heterogeneous TG regions, custom scripts were employed to annotate regions of interest (ROIs). We differentiated the bright DAPI signals of surrounding non-neuronal cells from the characteristically pale DAPI signals of sensory neurons [69]. These bright signals were excluded for further analysis, restricting our quantification strictly to TG neurons (examples shown in Supplementary Fig. 1). As dual RNAscope and IHC were adopted later in the study, specific cell markers were utilized to define cell types within the TNC and PAG, including protein markers for IHC (NeuN+ or Iba-1+) and mRNA probes for RNAscope ISH (*VGlut2* or *VGlut3* or *VGat*).

Anatomical localization of eCB metabolic gene expression in specific regions of interest was characterized by a semi-quantitative method. Furthermore, because the transcript abundance of some eCB metabolizing enzymes was highly variable in the TG (ranging from <10 transcript puncta in some neurons to > 60 in others), a semi-quantitative histological score (H-score) was calculated. The H−score was calculated using the following formula: ∑(n×%cells with score n). To ensure a sufficient cell count for robust quantification of H-score, 2 to 3 sections from a naive mouse brain were used per biological replicate (*n* = 2–3 section/mouse; *N* = 3–4 mice). The score binning strategy (0–3 or 0–4 scale) was adjusted based on the maximum transcript density and variability observed for each gene within a region. For example, for enzymes with high transcript abundance in TG neurons, a 0–4 bin scale was used (maximum possible score = 400), where the scoring criteria were defined as score 0 (cells with no puncta); score 1 (1–3 puncta); score 2 (4–9 puncta); score 3 (10–15 puncta); score 4 (>15 puncta). Finally, in TG, data from the V1 (ophthalmic) and V2 (maxillary) subdivisions were pooled for analyses due to the lower number of neurons identified in these regions compared to the V3 (mandibular) branch.

### Quantitative RT-PCR

Gene expression in specific regions of interest was assessed using quantitative RT-PCR (qPCR) following mTBI (*n* = 5–6/group). Fresh-frozen samples from the TNC and PAG were collected from 250 µm cryosections using the Palkovits punch technique. Total RNA was extracted using a Zymo Research spin column, following the manufacturer’s instructions. Due to the heavy myelination of TG samples, a 2-fold volume of TRI Reagent was utilized to ensure a sufficient RNA yield. RNA (0.6–1 μg) was then reverse transcribed using the Maxima First Strand cDNA Synthesis Kit, and qPCR was performed using the primer sets listed in Supplementary Table 2.

### Statistical analyses

Differences in the distribution of major enzyme transcripts within the AEA and 2-AG metabolic pathways were assessed using the non-parametric Kruskal-Wallis rank sum test. This was followed by post-hoc evaluation of pairwise differences in distribution shape using the Kolmogorov-Smirnov test to measure the distributional difference (D). Divergence intervals were identified using empirical cumulative distribution functions (eCDFs). A general linear model (GLM) was used to assess relationships (correlations) between puncta counts of synthesizing and hydrolyzing enzymes across different subdivisions of the TG, TNC and PAG, where the rate of change or divergence between different subdivisions was analyzed using an interaction term. ISH semi-quantitative H-scores from different cell populations and different subregions of sham and mTBI groups were analyzed using mixed-effect models. Due to the small sample size used in comparison between regions, Hedges’ g correction factor was also applied to quantify the effect size between two groups. Relative gene expression qPCR data between the sham and mTBI groups were tested using linear models (lm() function) on log transformed fold change. Righting reflexes were analyzed using a linear mixed-effect model (lmer() function) and pairwise differences between groups on each day were evaluated using estimated marginal means. Datasets were tested for normality assumptions and when assumptions were violated, a non-parametric test (Wilcoxon test) was used. Unless otherwise specified, effects were considered significant at an alpha level of 0.05. All analyses were performed using R 4.2.3 [70]. Packages from the tidyverse library were used for data processing and analyses [71]. All data were represented as mean ± standard error of the mean (SEM).

## Results

### Peripheral expression of AEA and 2-AG metabolizing enzymes

#### AEA metabolizing enzymes

**Fig. 1:**
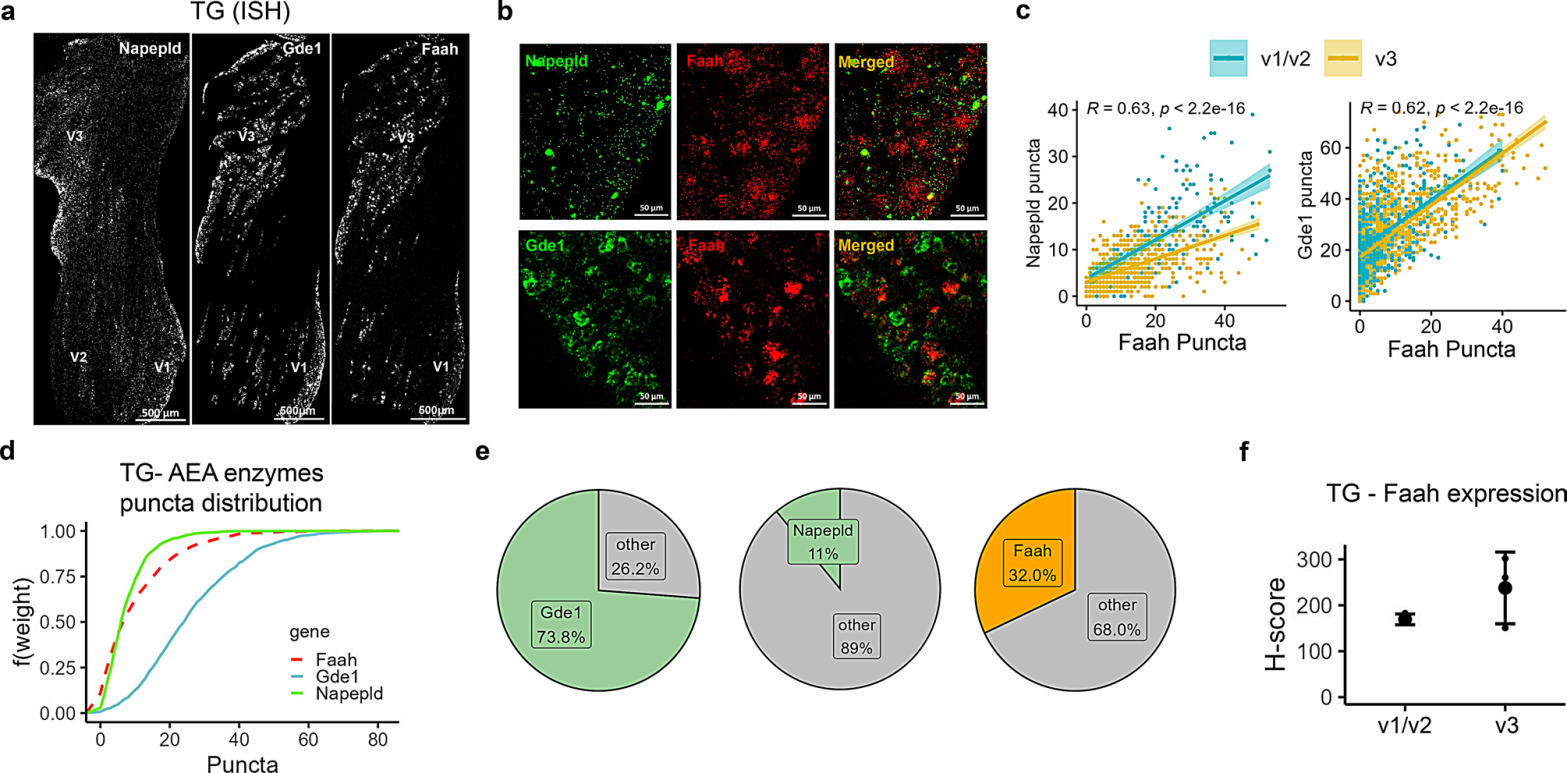
Spatial distribution of AEA-metabolizing enzyme transcripts in neurons of the trigeminal ganglion (TG). (**a**) Representative images illustrating the distribution of *Napepld*, *Gde1* and *Faah* in the three branches of a TG section obtained using RNAscope *in-situ* hybridization (ISH). (**b**) Images depicting co-labelling of the AEA synthesizing enzyme (*Napepld* or *Gde1*) and AEA hydrolyzing enzyme (*Faah*) in TG neurons. (**c**) Correlation graphs representing AEA metabolizing enzyme puncta counts illustrate the relationship between *Napepld* and *Faah*, as well as *Gde1* and *Faah*, within the three branches of TG (ophthalmic [V1]/maxillary [V2] (blue), and mandibular [V3] (yellow). (**d**) Empirical cumulative distribution function (ECDF) of AEA synthesizing and hydrolyzing enzyme puncta in TG cells with the following abundance in the expression of the enzymes: *Gde1*>*Faah*>*Napepld*. (**e**) Pie charts representing the proportion of TG neurons expressing AEA synthesizing (*Napepld* or *Gde1*) or hydrolyzing (*Faah*) enzyme transcripts. The total cell counts used in quantifying the proportion of neurons expressing each gene were: *Gde1* (*n* = 1716), *Napepld* (*n* = 803), and *Faah* (*n* = 803). (**f**) Comparison of H-score of the AEA hydrolyzing (*Faah*) enzyme between the primary branches (V1/V2 vs V3) of TG (mean ± SEM, *n* = 3 mice/region). H-score is the weighted histological score that circumvents the issue with the quantification of high- and low-expressing cells

We found that the AEA-metabolizing enzymes are present in all three subdivisions of the TG, albeit with varying distribution (Fig. 1a and b). A striking difference was observed in the co-expression of AEA-synthesizing enzymes (*Napepld* and *Gde1*) with the hydrolyzing enzyme (*Faah*) across these subdivisions. Specifically, a significantly higher number of neurons expressed both *Napepld* and *Faah* puncta in the V1/V2 compared to the V3 subdivision (Fig. 1c, *p* < 0.001). In contrast, a high number of neurons co-expressed *Gde1* and *Faah* across all three subdivisions (V1/V2/V3).

Although the widely studied AEA-synthesizing enzyme, *Napepld*, was present throughout the TG, its expression was low compared to other AEA-metabolic enzymes (*Gde1* > *Faah* > *Napepld*, Fig. 1d). Notably, *Faah* and *Napepld* shared a similar distribution profile (D = 0.155, *p* < 0.0001), whereas *Faah* and *Gde1* showed greater divergence (D = 0.5128, *p* < 0.0001). Of the 803 neurons identified, only 11% of the neurons expressed *Napepld* (Fig. 1e). Conversely, expression of the less-studied *Gde1* was abundant in the TG compared to *Napepld*; approximately three-fourths of TG neurons (~73.8% of 1716 neurons) expressed *Gde1* puncta (Fig. 1e).

Regarding the hydrolyzing enzymes, only about 32% of the 803 sensory neurons expressed *Faah* puncta in the TG (Fig. 1e), and no difference was noted among the different branches (Fig. 1f, *p* = 0.207). The percentage of TG neurons expressing eCB enzymes shared some similarities with the data found in the TG atlas obtained from snRNA-seq [72]: ~7% expressed *Napepld*, 80.7% expressed *Gde1*, and 20.8% of neurons expressed *Faah* (Supplementary Fig. 2a). Interestingly, we also detected AEA-metabolizing enzyme expression in the TREZ, with a relative abundance of *Faah* > *Napepld* > *Gde1* (Fig. 3a, c and d).

#### 2-AG metabolizing enzymes

**Fig. 2:**
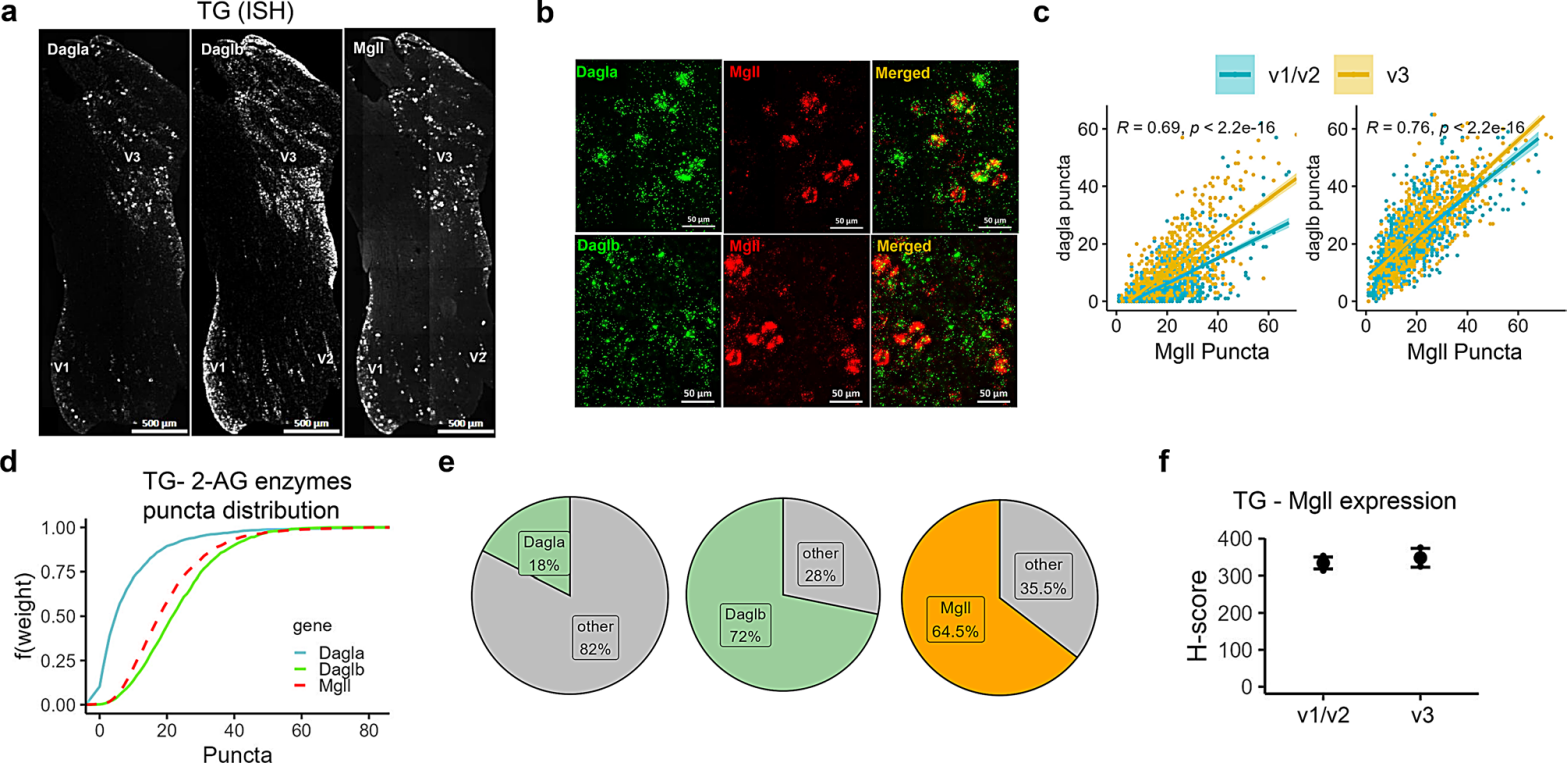
Spatial distribution of 2-AG metabolizing enzyme transcripts in neurons of the trigeminal ganglion (TG). (**a**) Representative ISH images illustrating the distribution of *Dagla*, *Daglb* and *Mgll* in the three branches of a TG section. (**b**) Images showing co-labelling of the 2-AG synthesizing enzyme (*Dagla* or *Daglb*) and 2-AG hydrolyzing enzyme (*Mgll*) in TG neurons. (**c**) Correlation graphs representing 2-AG metabolizing enzyme puncta counts show the relationship between *Dagla* and *Mgll* as well as *Daglb* and *Mgll* in three branches (ophthalmic [V1]/maxillary [V2] (blue), and mandibular [V3] (yellow). (**d**) Empirical cumulative distribution function (ECDF) of 2-AG synthesizing and hydrolyzing enzyme puncta in TG cells where *Daglb*>*Mgll*>*Dagla*. (**e**) Pie charts representing the proportion of TG neurons expressing 2-AG synthesizing (*Dagla* or *Daglb*) or hydrolyzing (*Mgll*) enzyme transcripts. The proportion of *Dagla*, *Daglb* and *Mgll* transcript-containing neurons was quantified from a total of *n* = 2022 TG neurons. (**f**) Comparison of H-score of 2-AG hydrolyzing (*Mgll*) enzyme between the primary branches (V1/V2 vs V3) of TG (*n* = 3 mice/region). H-score is the weighted histological score, and data are presented as mean ± SEM

Similar to AEA-metabolizing enzymes, 2-AG metabolizing enzymes were also present throughout TG, albeit with differences in the expression of their synthesizing enzymes (Fig. 2a). Both *Dagla* and *Daglb* were present in sensory neurons throughout the TG, and both co-localize with *Mgll*, though with varying expression levels (Fig. 2b and c). Specifically, the expression of *Dagla* and *Mgll* puncta was higher in cells of the V3 branch of the TG compared to the V1/V2 branches (Fig. 2c, *p* < 0.001). Among the metabolizing enzymes, *Daglb* was highly expressed in the TG (Fig. 2d). Here, *Daglb* and *Mgll* shared a similar distribution pattern (D = 0.1355, *p* < 0.0001), whereas *Dagla* and *Mgll* showed greater divergence (D = 0.5163, *p* < 0.0001).

**Fig. 3:**
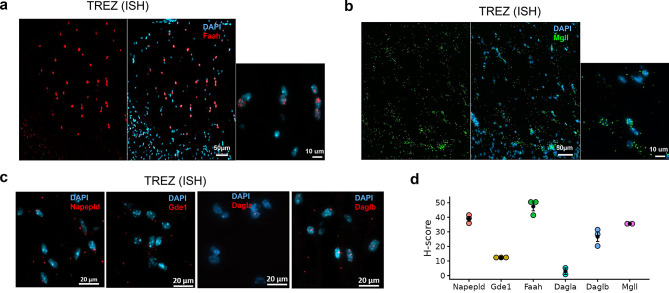
Distribution of major eCBs metabolizing enzymes in the trigeminal root entry zone (TREZ). (**a**–**c**) Representative ISH images from a TREZ show expression of hydrolyzing enzymes *Faah* (**a**), *Mgll* (**b**), and synthesizing enzymes *Napepld*, *Gde1*, *Dagla*, and *Daglb* (**c**). (**d**) Comparison of H-scores among synthesizing and hydrolyzing enzymes in the TREZ. H-score is the weighted histological score, and ISH data (H-score, *n* = 3 mice tissues/gene type) are presented as mean ± SEM

A total of 2022 neurons were quantified in the TG to assess 2-AG synthesizing enzyme transcript expression. Only about 18% of the neurons expressed *Dagla*. In contrast to *Dagla*, but like *Gde1* for AEA synthesis, about 72% of TG neurons expressed *Daglb*. Unlike *Faah*, almost 65% of neurons in the TG expressed *Mgll* (Fig. 2e), and no difference was observed in the expression of *Mgll* across the different TG subdivisions (Fig. 2f). Except for *Dagla* (~15%), there is a discrepancy between our data and the TG atlas regarding the expression of *Mgll* (~47%) and *Daglb* (21%) in the TG neurons (Supplementary Fig. 2b). Similar to the AEA system, 2-AG metabolizing enzymes are also present in the TREZ, but at a lower proportion, following the pattern *Mgll* > *Daglb* > *Dagla* (Figs. 3b, c and d).

#### AEA and 2-AG cognate receptors in the TG

**Fig. 4:**
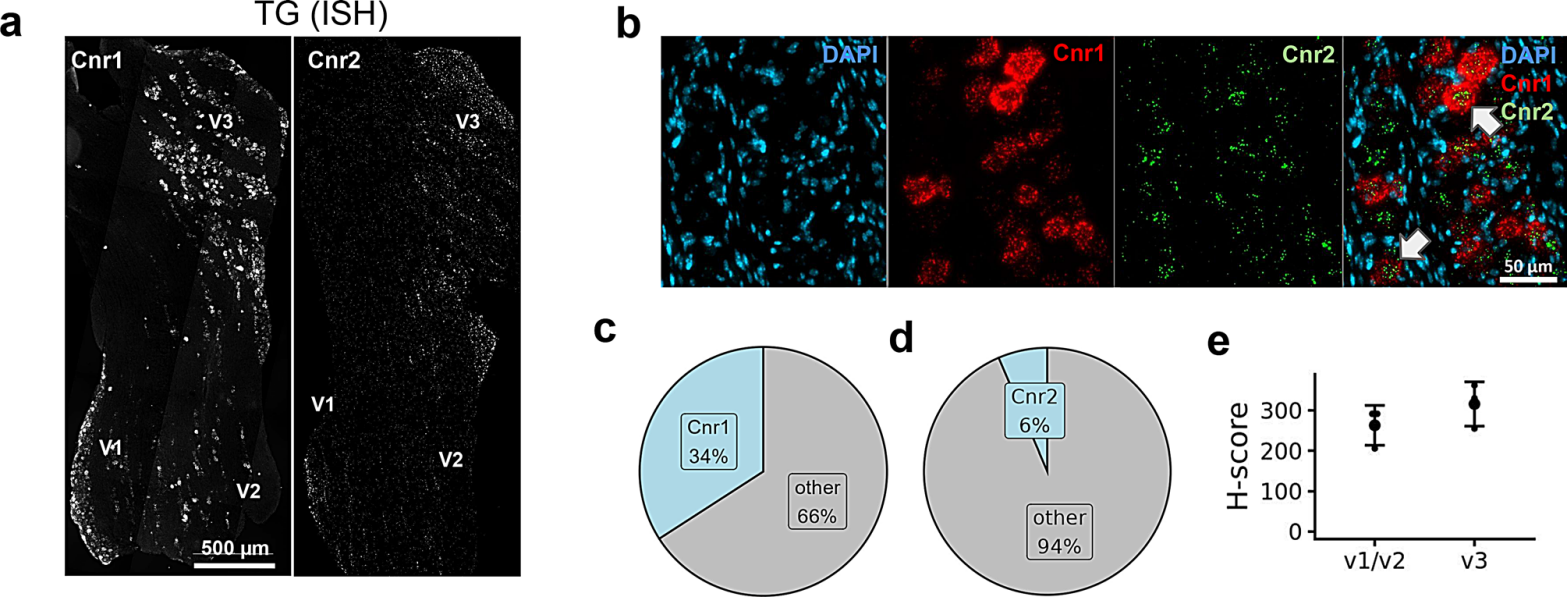
Spatial distribution of the *Cnr1* and *Cnr2* receptors in neurons of the trigeminal ganglion (TG). (**a**) ISH image panels depicting distribution of *Cnr1* and *Cnr2* in a TG section. (**b**) Magnified images showing co-labeling of *Cnr1* and *Cnr2* in TG neurons. Note the increased abundance of *Cnr1* (red) in the TG compared to *Cnr2* (green) which is occasionally present within *Cnr1* neurons (arrows). (**c**) Pie chart representing the proportion of TG neurons (*n* = 5190) expressing Cnr1 transcripts. (**d**) Pie chart representing the proportion of TG neurons (*n* = 5190) expressing Cnr2 transcripts. (**e**) Comparison of *Cnr1* H-score between the primary branches (V1/V2 vs V3) of TG (*n* = 3 mice/region). H-score is the weighted histological score, and data are presented as mean ± SEM

Consistent with existing literature [73], results from the present study showed that *Cnr1* expression was abundant in TG neurons (Fig. 4a and b). In contrast to a previous report showing the absence of Cnr2 expression in the rat TG [73], but in agreement with the mouse TG atlas [72], we found the *Cnr2* expression was confined to only a small number of neuronal cells (Fig. 4b and d). While a recent report observed *Cnr1* in 55% of TG neurons [41], we found that approximately one-third of TG neurons expressed *Cnr1*, a finding that aligns with both an earlier study [73] and the TG atlas [72]. Specifically, of the 5190 neurons quantified, approximately 34% expressed *Cnr1* transcripts (Fig. 4c). Although the mean *Cnr1* H-score appeared higher in the V3 subdivision, no significant differences were observed across the TG subdivisions (Fig. 4e, *p* = 0.2834). Finally, whereas Cnr2 was reported in about 1% of neurons in the TG atlas, here we found that Cnr2 is expressed in ~6% of TG neurons (Fig. 4d).

### Central expression of AEA and 2-AG metabolizing enzymes in the trigeminal nucleus caudalis (TNC)

**Fig. 5:**
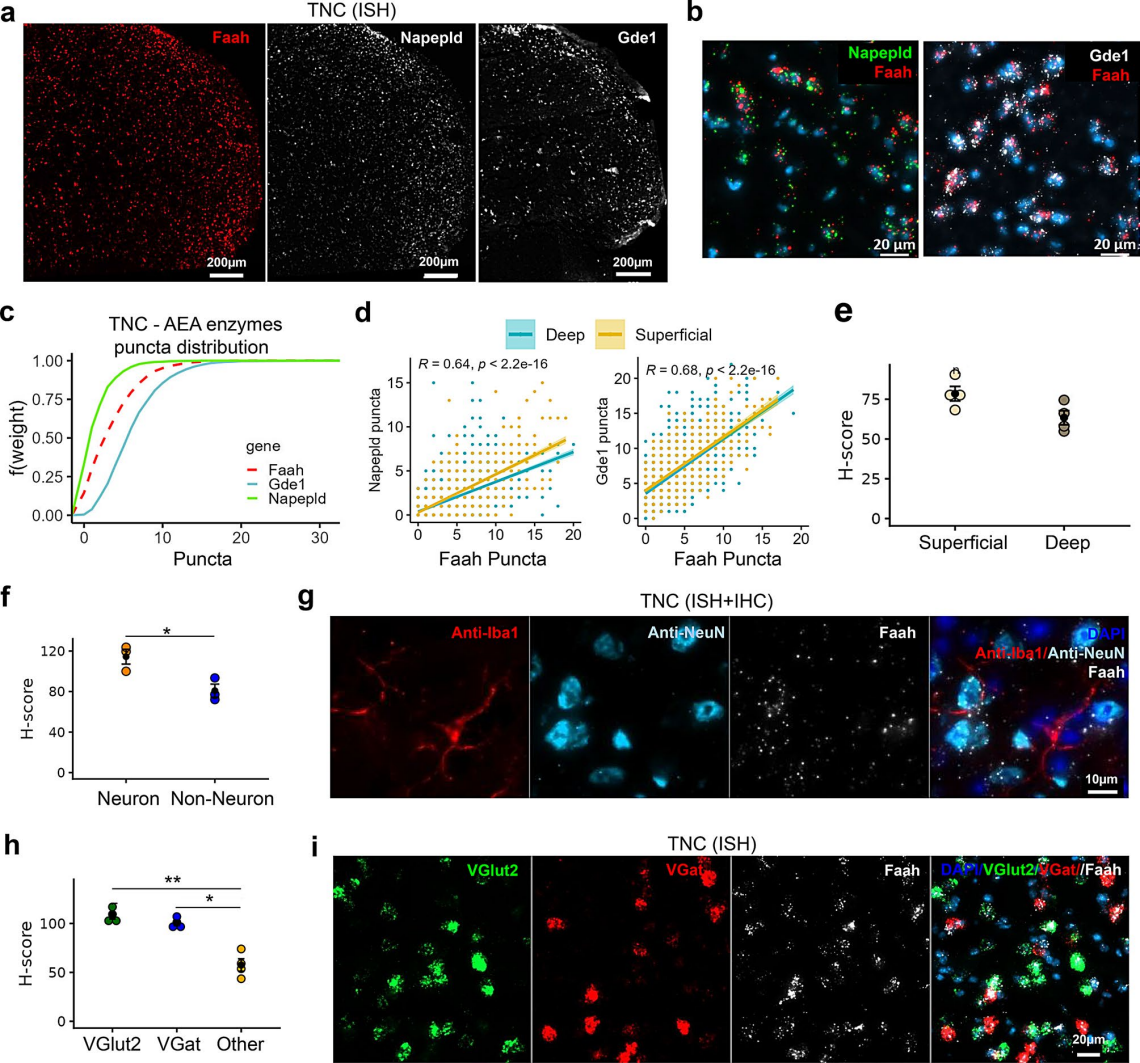
Spatial distribution of AEA metabolizing enzyme transcripts in trigeminal nucleus caudalis (TNC). (**a**) Representative ISH images illustrating the distribution of hydrolyzing enzyme (*Faah*) and synthesizing enzymes (*Napepld*/*Gde1*) in the TNC. (**b**) Images showing co-labeling of *Faah* and *Napepld* as well as *Faah* and *Gde1* within the same cells in the TNC. (**c**) Empirical cumulative distribution function (ECDF) of the synthesizing and hydrolyzing enzymes in the TNC shows the abundance of gene transcripts (*Gde1*>*Faah*>*Napepld*). (**d**) correlation graphs comparing AEA metabolizing enzyme puncta counts in the superficial (yellow) and deep (blue) layers of the TNC. (**e**) Comparison of *Faah* H-score present in neurons between superficial and deep layers of the TNC. (**f**) Comparison of *Faah* H-score between neuronal (NeuN+) and non-neuronal (DAPI) cells in the TNC. (**g**) Image showing increased presence of *faah* transcripts in neurons (NeuN+) compared to microglia (Iba-1+) in the TNC. (**h**) Comparison of *Faah* H-score among *VGlut2*+, *VGat*+ neurons and other cells (DAPI) in the TNC. (**i**) Image showing increased presence of *Faah* in *VGlut2*+ and *VGat*+ neurons than in DAPI cells in the TNC. H-score is the weighted histological score, and data are presented as mean ± SEM (*n* = 3–4 mice/region or cell type)

**Fig. 6:**
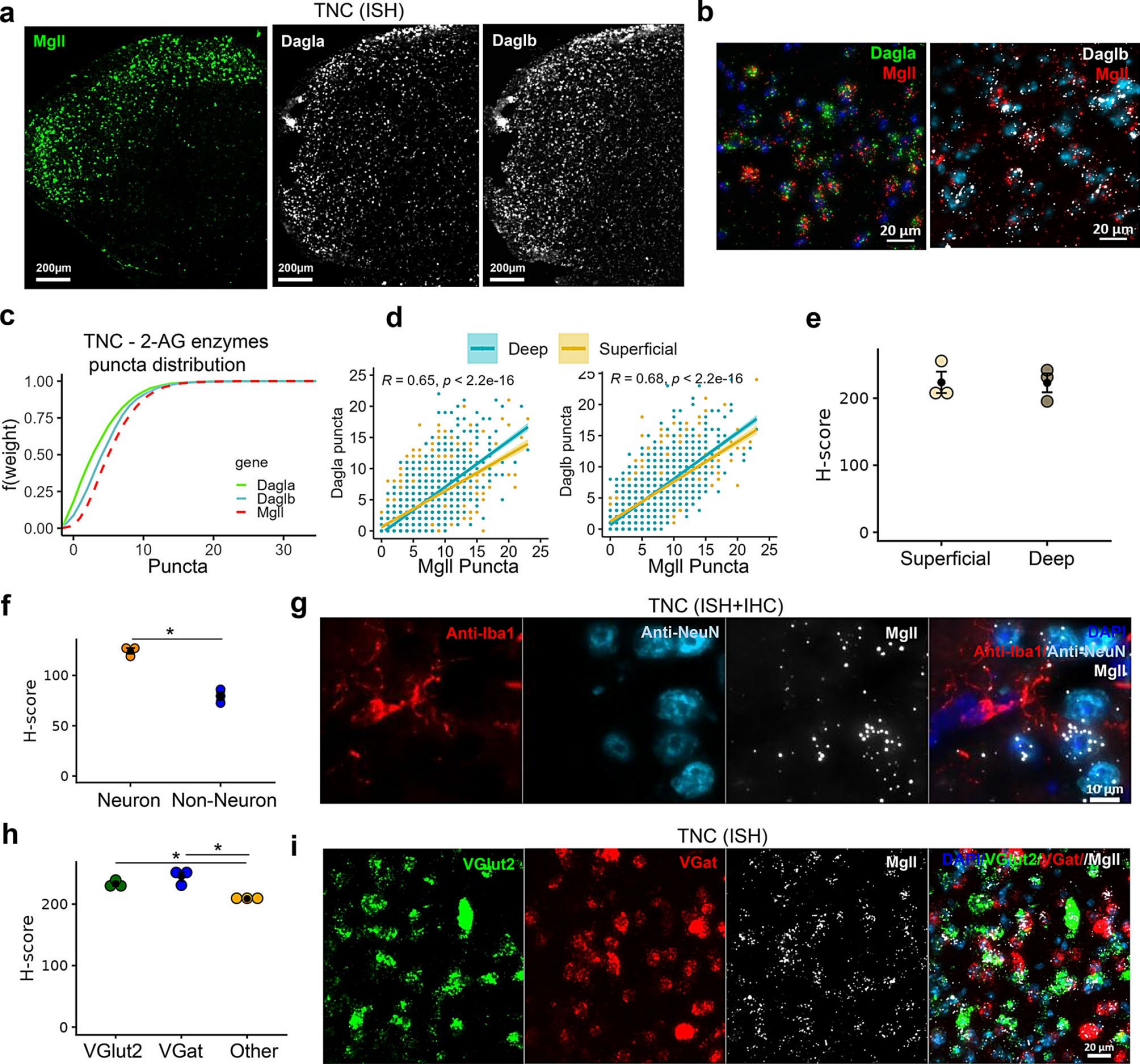
Spatial distribution of 2-AG metabolizing enzyme transcripts in trigeminal nucleus caudalis (TNC). (**a**) Representative ISH images showing the distribution of *Mgll*, *Dagla*, and *Daglb* in TNC. (**b**) Images showing co-labelling of *Mgll* and *Dagla* or *Mgll* and *Daglb* in cells within TNC. (**c**) Empirical cumulative distribution function (ECDF) depicts predominance for *Mgll* distribution compared to *Dagla* and *Daglb* (*Mgll*>*Daglb*>*Dagla*) in the TNC. (**d**) Correlation between 2-AG synthesizing enzymes (*Dagla* or *Daglb*) and 2-AG hydrolyzing enzyme (*Mgll*) puncta counts in the superficial (yellow) and deep (blue) layers of the brainstem TNC. (**e**) Comparison of *Mgll* H-score from neurons between superficial and deep layers of the TNC. (**f**) Comparison of *Mgll* H-score shows increased presence of *Mgll* in neurons (NeuN+) compared to non-neuronal (DAPI) cells in the TNC. (**g**) Image shows abundance of *Mgll* transcripts in neurons (NeuN+) compared to microglia (Iba-1+). (**h**) Dotplot represents increased *Mgll* H-score in the *VGlut2*+ neurons and *VGat*+ neurons compared to other cell types (DAPI). (**i**) An image panel shows co-localization of *Mgll* transcripts within *VGlut2*+ neurons and *VGat*+ neurons. The H-score data are presented as mean ± SEM (*n* = 3–4 mice/region or cell type)

The expression of AEA-metabolizing enzymes was remarkably lower in the brainstem TNC (Fig. 5) compared to the 2-AG metabolizing enzymes (Fig. 6). Akin to the expression pattern of AEA metabolizing enzymes in the TG, the TNC displayed the relative abundance of *Gde1* > *Faah* > *Napepld* (Fig. 5a–c). Although the majority of cells in the TNC co-expressed puncta of both AEA synthesizing enzymes (*Napepld* or *Gde1*) and hydrolyzing enzymes (*Faah*) (Fig. 5b), cells expressing *Napepld* and *Faah* shared a similar distribution of puncta (Fig. 5c, D = 0.2989, *p* < 0.0001) compared to those expressing *Gde1* and *Faah* (Fig. 5c, D = 0.3359, *p* < 0.0001).

Within the TNC, *Napepld*/*Faah* puncta co-expression was higher in cells of the superficial layer compared to the deep layers (Fig. 5d, *p* < 0.001). Furthermore, consistent with earlier immunohistochemical reports [74, 75], *Faah* expression was higher (though non-significantly) in the superficial layer of the TNC compared to the deep layers (Fig. 5e; *p* = 0.054; large effect size Hedges’ g = 1.68); its expression was strongly correlated with co-expression of *Napepld* and *Gde1* (Fig. 5d).

Of the 7080 cells (4012 neurons and 3068 non-neuronal cells) quantified in the TNC, approximately 56% were neurons. When compared between common neuronal elements (anti-NeuN) and non-neuronal cells (DAPI positive/NeuN negative), *Faah* expression was significantly higher in neurons (*p* = 0.026; large effect size Hedges’ g = 2.81) than non-neuronal cells (Fig. 5f). Since our previous study found that microglia were more abundant than astrocytes in the deep layers of TNC [19], we specifically compared *Faah* presence in neurons (NeuN+) versus microglia (Iba-1+) cells. Consistent with the earlier observation, *Faah* puncta were abundant in neurons, while only sparse labeling was noted in Iba+ microglia (Fig. 5g). Furthermore, *Faah* expression was assessed in the *VGlut2*+ and *VGat*+ neurons (Fig. 5h and i). Within these neuronal populations, Faah was expressed at similar levels in both neuronal types and at higher levels than in other cell types (*p* < 0.0001).

In the TNC, gene expression of the major 2-AG metabolizing enzymes was abundant compared to the expression of AEA metabolizing enzymes (Fig. 6). Both *Dagla* and *Daglb* were equally present in the TNC and co-expressed with *Mgll* (Fig. 6b and d), following the expression pattern *Mgll* > *Daglb* > *Dagla* (Fig. 6c). Cells in the TNC shared a slightly higher co-distribution profile of *Daglb* and *Mgll* puncta (Fig. 6d, D = 0.1198, *p* < 0.0001) compared to *Dagla* and *Mgll* (Fig. 6d, D = 0.2651, *p* < 0.0001).

In contrast to *Faah*, no differences were observed in the *Mgll* expression between the superficial and deep layers of the TNC (Fig. 6e, *p* = 0.544). However, *Dagla* expression was higher in the superficial layer compared to the deep layers (Supplementary Fig. 3a; p < 0.01; large effect size Hedges’ g = 4.68). The presence of *Mgll* in neurons and non-neuronal cells was also compared. Similar to *Faah*, *Mgll* expression was significantly higher in neurons (Fig. 6f; *p* < 0.001; large effect size Hedges’ g = 6.06), with sparse labeling in microglia (Fig. 6g). Likewise, *Mgll* was higher in *VGlut2*+ and *VGat*+ neurons than in other cell types (Fig. 6h and i, *p* < 0.05).

### AEA and 2-AG cognate receptors in the TNC

**Fig. 7:**
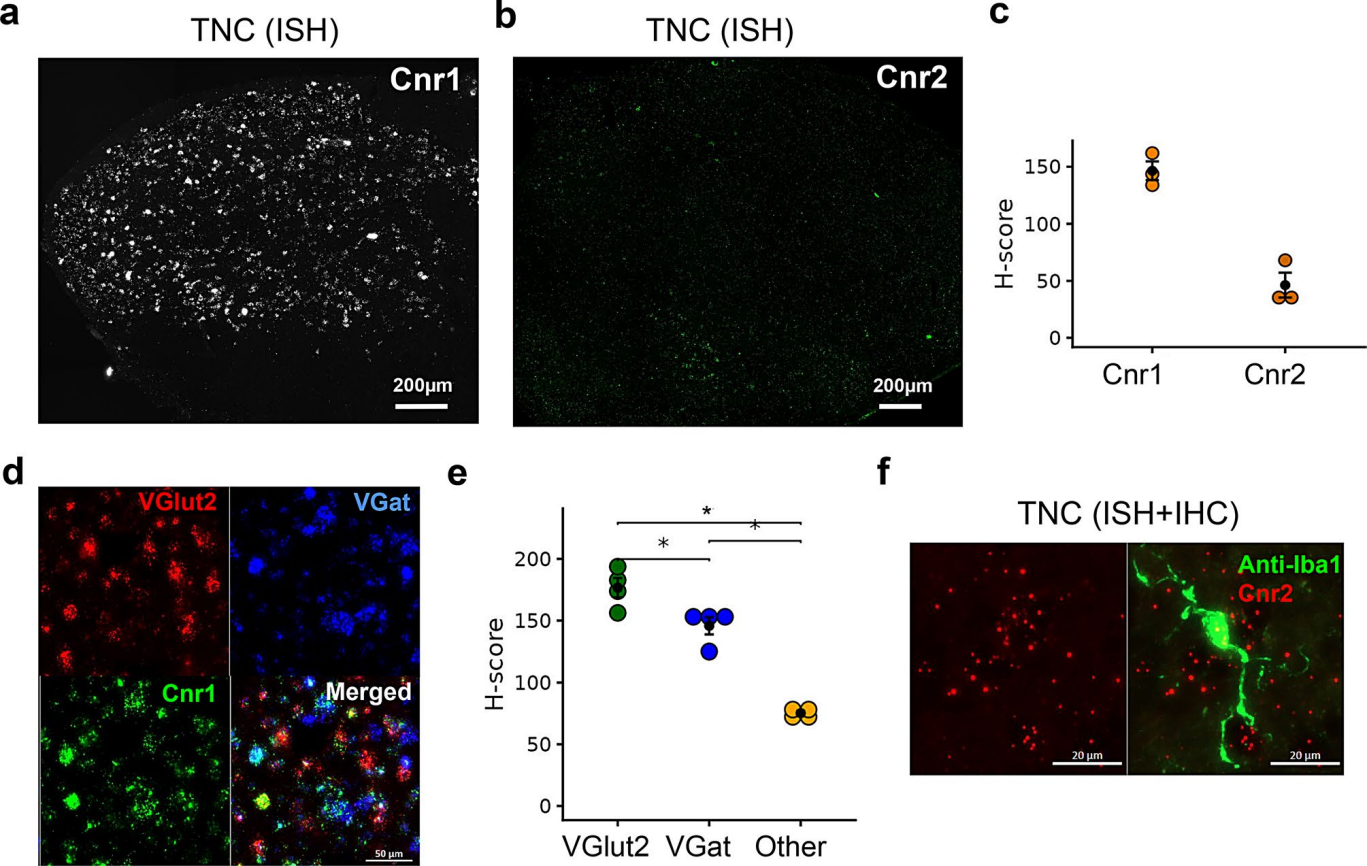
Distribution of eCB cognate receptors in the TNC. ISH images representing *Cnr1* (**a**) and *Cnr2* (**b**) expressions in TNC. (**c**) Comparison of H-score between *Cnr1* and *Cnr2* indicates the predominance of *Cnr1* in the brainstem TNC. (**d**) ISH image representing the co-labelling of *Cnr1* in *VGlut2*+ and *VGat*+ neurons in the TNC. (**e**) Comparison of H-scores shows increased expression of *Cnr1* in the *VGlut2*+ compared to *VGat*+ neurons or other cell types in the TNC. (**f**) ISH (*Cnr2*, red) and immunolabeling of microglia (Iba-1+, green) show sparse *Cnr2* transcripts present in microglial cells (see also Supplementary Fig. 3b). The H-score data are presented as mean ± SEM (*n* = 3–4 mice/gene type or cell type)

Similar to earlier reports [76], one of the primary receptors for eCBs, *Cnr1*, was present throughout the superficial and deep layers of the TNC (Fig. 7a) and was predominantly located in neurons. Within the TNC, *Cnr1* expression was significantly higher in the *VGlut2* neurons compared to *VGat* neurons (Fig. 7d and e, *p*-value = 0.019). Of the 983 neurons quantified in the TNC, approximately 25% of *VGlut2*+ neurons co-expressed *Cnr1*, while ~9% *VGat*+ neurons co-expressed *Cnr1*. Consistent with existing literature (see [77]), only sparse labelling of *Cnr2* was detected throughout the TNC compared to *Cnr1* (Fig. 7b and c). Since *Cnr2* is known to be expressed in microglia in specific brain regions, we also assessed *Cnr2* puncta expression in the brainstem microglial population using anti-Iba-1. Although Iba-1+ cells express *Cnr2* transcripts in the TNC, *Cnr2* expression is not exclusive to microglia (Fig. 7f). Further, no difference was observed between Iba-1+ cells and other DAPI-identified cells in the TNC (Supplementary Fig. 3b).

**Fig. 8:**
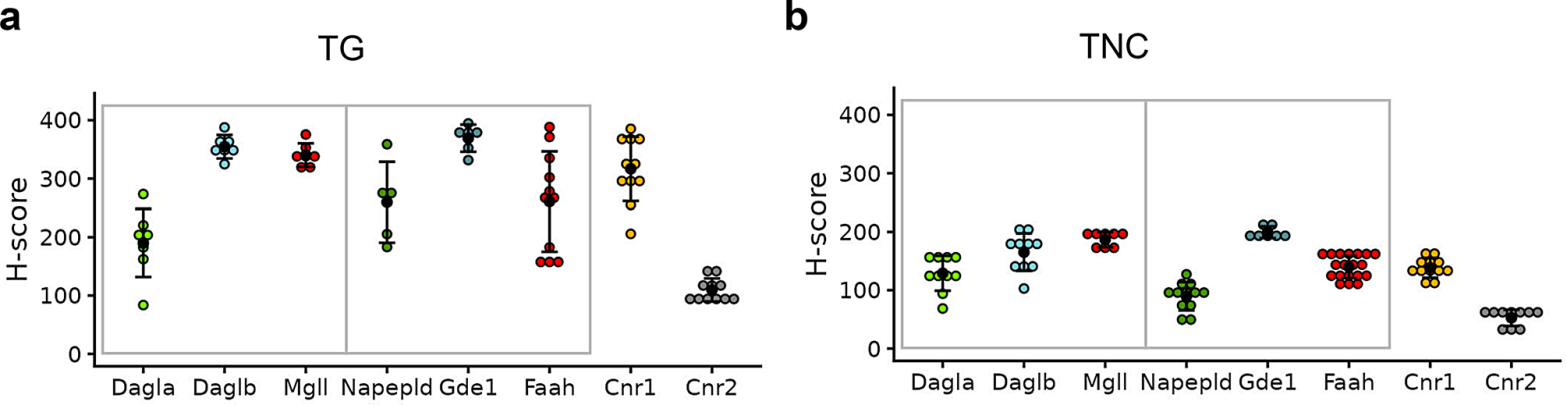
Comparison of the H-scores of eCB metabolizing enzymes and eCB cognate receptors in the peripheral trigeminal ganglion (TG) (**a**) and brainstem trigeminal nucleus caudalis (TNC) (**b**). Note the high variability in TG compared to TNC, reflecting the heterogeneity of the TG cell population. Each dot represents the H-score derived using DAPI positive cells from a single tissue section. *n* = 2–4 sections/mouse; *N* = 3–4 mice. Notably, *Cnr1* H-score is comparable to those of the eCB hydrolyzing enzymes (*Faah*/*Mgll*) in both the TG and the TNC

Overall, within the trigeminal complex, the expression of AEA and 2-AG hydrolyzing enzymes was higher in the TG sensory neurons compared to cells in the TNC (Fig. 8), with a mean H-score of > 250 in the TG and <200 in the TNC. When comparing the TG and TNC within the trigeminal complex, all genes assessed in the TG exhibited higher variability (Fig. 8a), likely due to the heterogeneous population of cells in the ganglion (see below in Fig. 10). Among the 2-AG metabolizing enzymes, the mean H-score for *Dagla* was low in both the TG (~200) and TNC (~120) (Fig. 8a and b). Similarly, among the AEA-metabolizing enzymes, the mean H-score for *Napepld* was lower in the TG (~250) and TNC (~95) compared to *Gde1* and *Faah*. Interestingly, the mean H-score for *Cnr1* was similar to that of the AEA and 2-AG hydrolyzing enzymes, whereas *Cnr2* was expressed at low levels.

### TBI induced behavioral changes

**Fig. 9:**
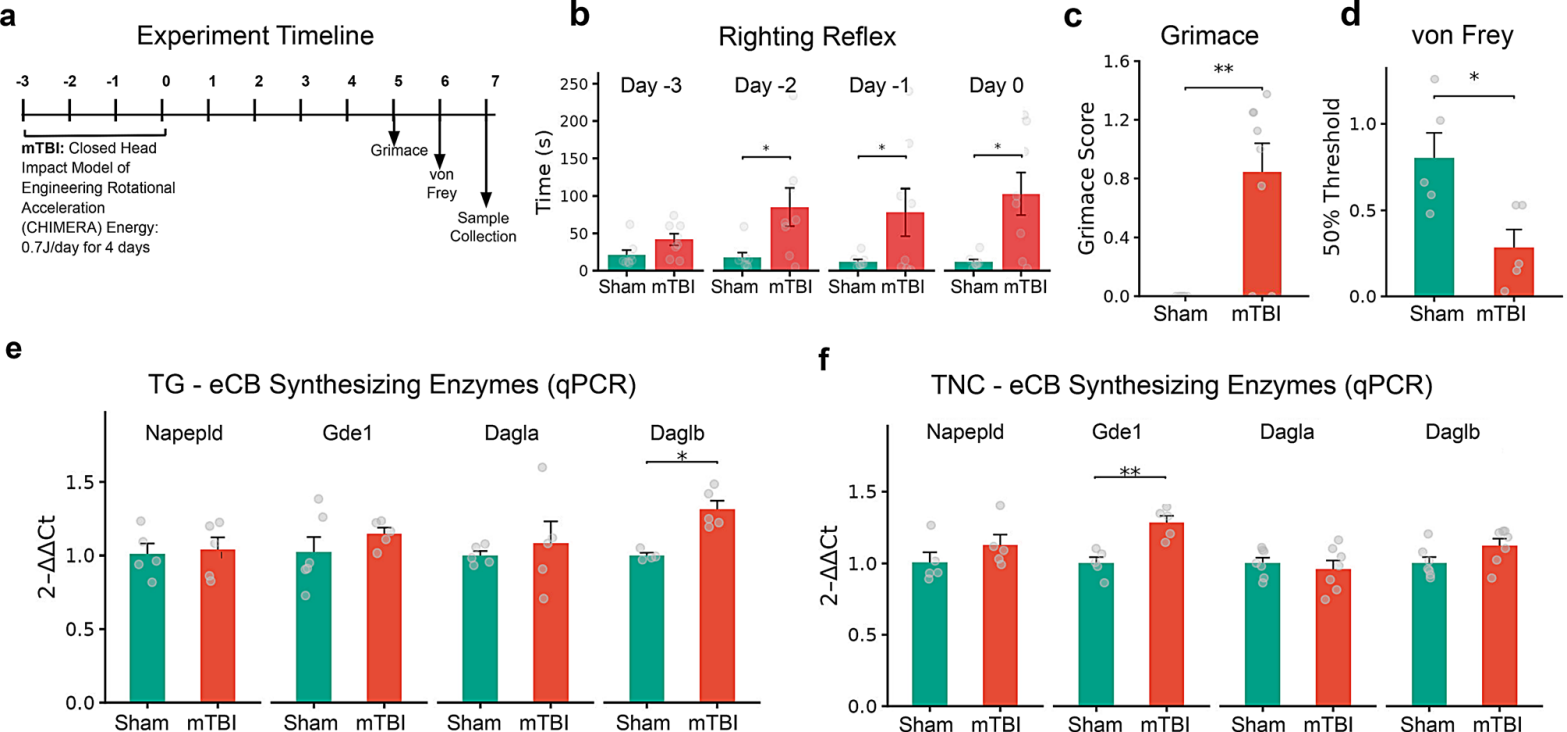
Experimental timeline, behavioral assessments, and mTBI-induced changes in major eCB synthesizing enzymes in the trigeminal complex. (**a**) A schematic of the repetitive mTBI protocol, behavioral assessments and sample collection timeline. (**b**) Comparison of righting reflex (duration) between sham and mTBI groups. (**c**) Average mouse grimace scale (MGS) score for sham and mTBI groups at 5 days post-injury. (**d**) Comparison of periorbital tactile sensitivity measured using von frey filaments at 6 days post-injury. (**e**, **f**) Relative gene expressions (qPCR) of synthesizing enzymes in the TG (**e**) and the TNC (**f**) 7 days following mTBI. *n* = 5–7 mice/group. Data are presented as mean ± SEM (* *p* < 0.05, **p < 0.01, *** *p* < 0.001, and **** *p* < 0.0001)

The repetitive mTBI induced neurobehavioral pathologies similar to our earlier reports [18, 19]. Each of the four repetitive mTBI injuries prolonged the time to righting reflex, a measure of the duration of unconsciousness following injury. Although the injury on the first impact (day −3) did not induce a significant difference between the sham and mTBI groups (Fig. 9b, *p* = 0.428), injuries on subsequent days (day −2, day −1 and day 0) significantly increased the righting reflex latency in the mTBI group (Fig. 9b, *p* < 0.05). Notably, the average righting reflex time in mTBI mice was approximately 74 s longer than that of the sham group (day −2 = 66.9s; day −1 = 65.6s; day 0 = 90.8s).

Five days following the last mTBI, we assessed spontaneous cephalic pain responses. Compared to sham controls, mTBI mice exhibited increased orbital tightening and altered ear position. Accordingly, the grimace scores (MGS) of the mTBI group were significantly higher than that of the sham group (Fig. 9c, *p* < 0.01). We also observed that the mTBI mice exhibited a significantly reduced peri-orbital tactile threshold six days following mTBI compared to their sham counterparts (Fig. 9d, *p* < 0.05).

### TBI induced changes in the gene expression of AEA and 2-AG hydrolyzing enzymes in the trigeminal complex

Global gene expression changes following mTBI were analyzed using qPCR, followed by an assessment of cell- and region- specific spatial changes using ISH. Considering the expression of the enzymes was widely distributed throughout the TG, including sensory neurons, fiber tracts, and the trigeminal root entry zone (TREZ), we analyzed and compared expression patterns in these regions between the sham and mTBI group 7 days after the last repetitive mTBI. Except for *Daglb* in the TG (qPCR - Fig. 9e, *p* = 0.0012) and *Gde1* in the TNC (qPCR - Fig. 9f, *p* < 0.01), no significant changes were observed for the other major synthesizing enzymes following mTBI. Within the TNC, ISH also showed no significant differences in the expression of synthesizing enzymes between the groups (Supplementary Fig. 3c–f).

**Fig. 10:**
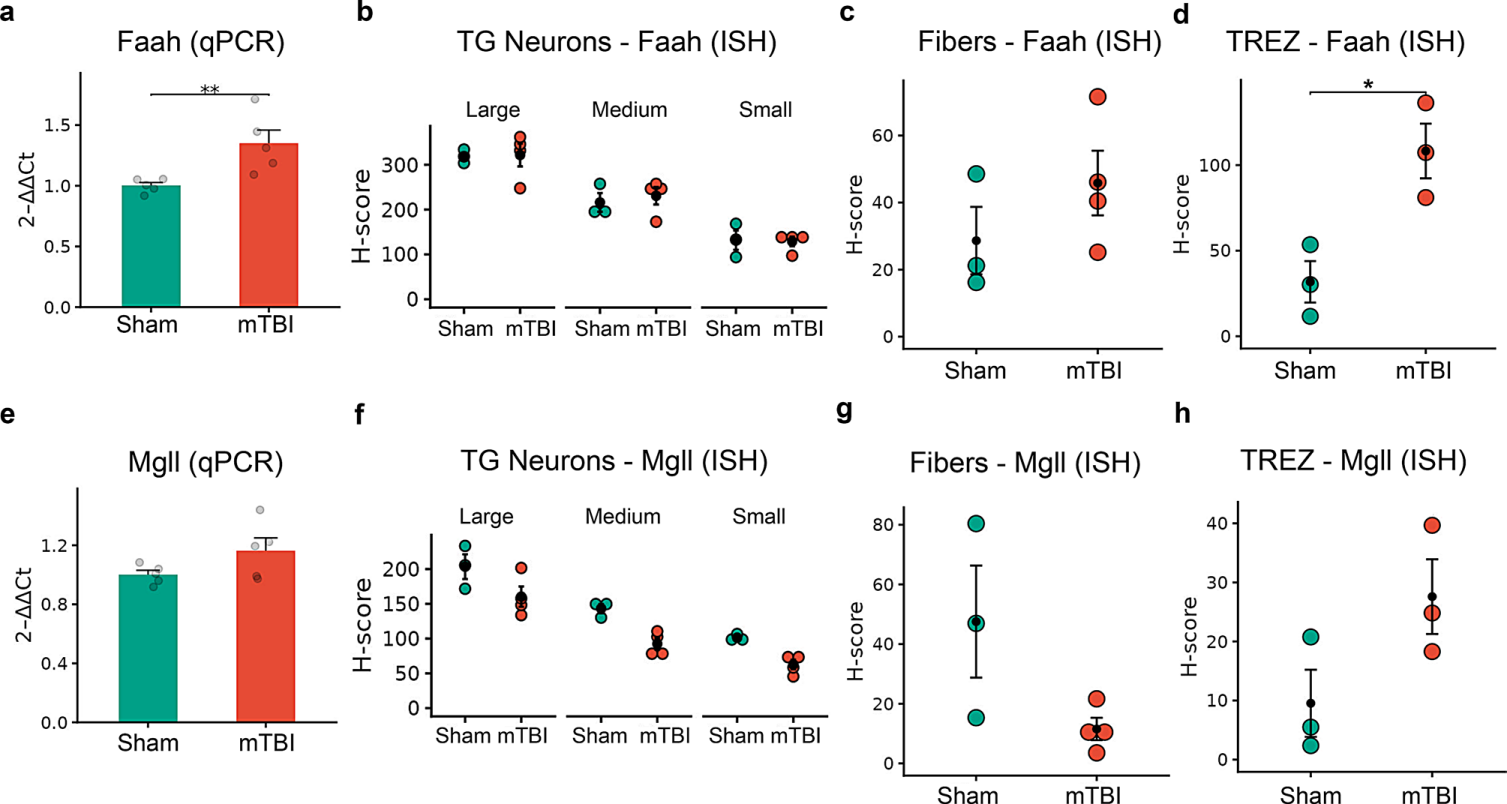
mTBI-induced changes in the gene expression of eCB hydrolyzing enzymes (*Faah*/*Mgll*) present in the trigeminal ganglion (TG). (**a**) Comparison of relative expression (qPCR) of *Faah* from the whole TG between sham and mTBI groups. (**b**-**d)**. Dot plots comparing *Faah* H-score (ISH) between sham and mTBI, from (**b**) 3 morphometric classes (large, medium and small) of TG neurons, (**c**) fibers within the TG and (**d**) trigeminal root entry zone (TREZ). (**e**) Relative expression (qPCR) of *Mgll* between sham and mTBI groups. (**f**-**h)** Comparison of ISH *Mgll* H-score between sham and mTBI groups in the TG neurons (**f**), fibers in the TG (**g**) and TREZ (**h**). Relative expression data (bar graphs) are presented as mean ± SEM (*n* = 5–7 mice/group). Dot plots representing H-scores are presented as mean ± SEM (*n* = 3–4 mice/group). * *p* < 0.05, **p < 0.01, *** *p* < 0.001 and **** *p* < 0.0001

In the TG, qPCR revealed that *Faah,* but not *Mgll,* was significantly upregulated in the mTBI group (Fig. 10a: *Faah* - *p* < 0.01; Fig. 10e: *Mgll* - *p* = 0.109). Unexpectedly, however, ISH analysis showed no difference in *Faah* (*p* = 0.99, Fig. 10b) or *Mgll* (Fig. 10f) expression within the 3 morphometric classes of TG sensory neurons. Similarly, in TG fibers, where DAPI stains expressed eCB enzyme transcripts, there was no difference in *Faah* (Fig. 10c, *p* = 0.27), though a non-significant decrease in *Mgll* was observed (Fig. 10g, *p* = 0.07). Notably, we found one week post-mTBI, both AEA and 2-AG hydrolyzing enzymes *Faah* (Fig. 10d, p < 0.05, large effect size Hedges’ g = 3.11) and *Mgll* (Fig. 10h, *p* = 0.101, large effect size Hedges’ g = 1.73) were elevated in the TREZ, a region predominantly populated by glial cells (Supplementary Fig. 4).

**Fig. 11:**
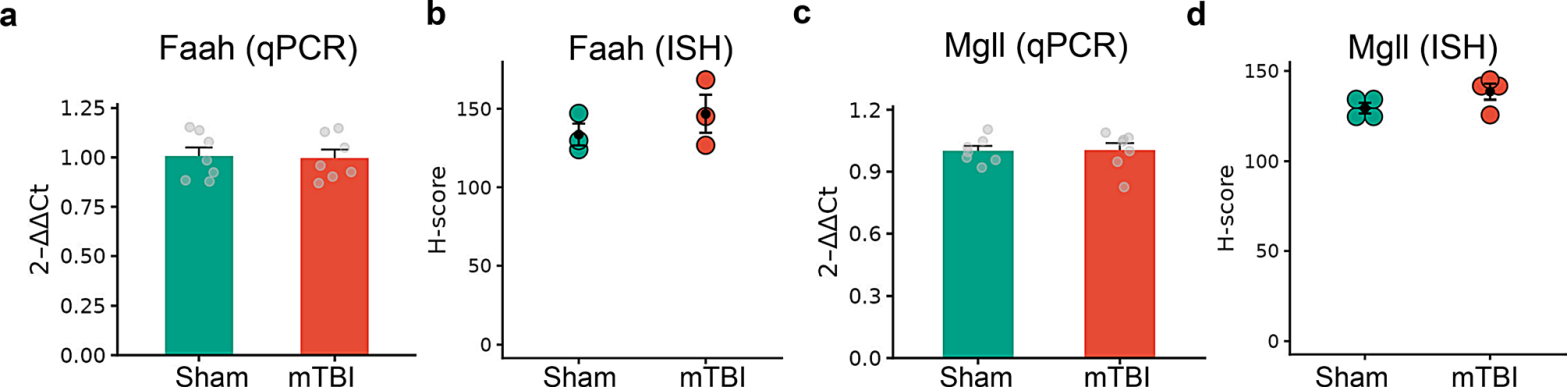
mTBI-induced changes in the gene expression of major eCB hydrolyzing enzymes in the trigeminal nucleus caudalis (TNC). **a, b** Comparison of *Faah* gene expression in the TNC between sham and mTBI groups assessed by qPCR (**a**) and ISH (**b**). **c, d** Comparison of *Mgll* expression in the TNC between sham and mTBI groups using qPCR (**c**) and from ISH (**d**). qPCR (relative expression, *n* = 5–6 mice/group) and ISH data (H-score, *n* = 3–4 mice/group) are presented as mean ± SEM

Within the TNC, mTBI did not induce significant changes in AEA or 2-AG metabolizing enzymes as measured by qPCR (Fig. 11a and c). However, ISH data showed a trend towards increased *Faah* (Fig. 11b, *p* = 0.1131) and *Mgll* (Fig. 11d, *p* = 0.1419) expression in the TNC neurons (Hedges’ g = 1.2 and 1.1, respectively). This difference was not observed in the microglial population between the groups or when DAPI was used as a proxy to quantify non-neuronal cells (Supplementary Fig. 3g and h).

### AEA and 2-AG metabolizing enzymes in the midbrain

**Fig. 12:**
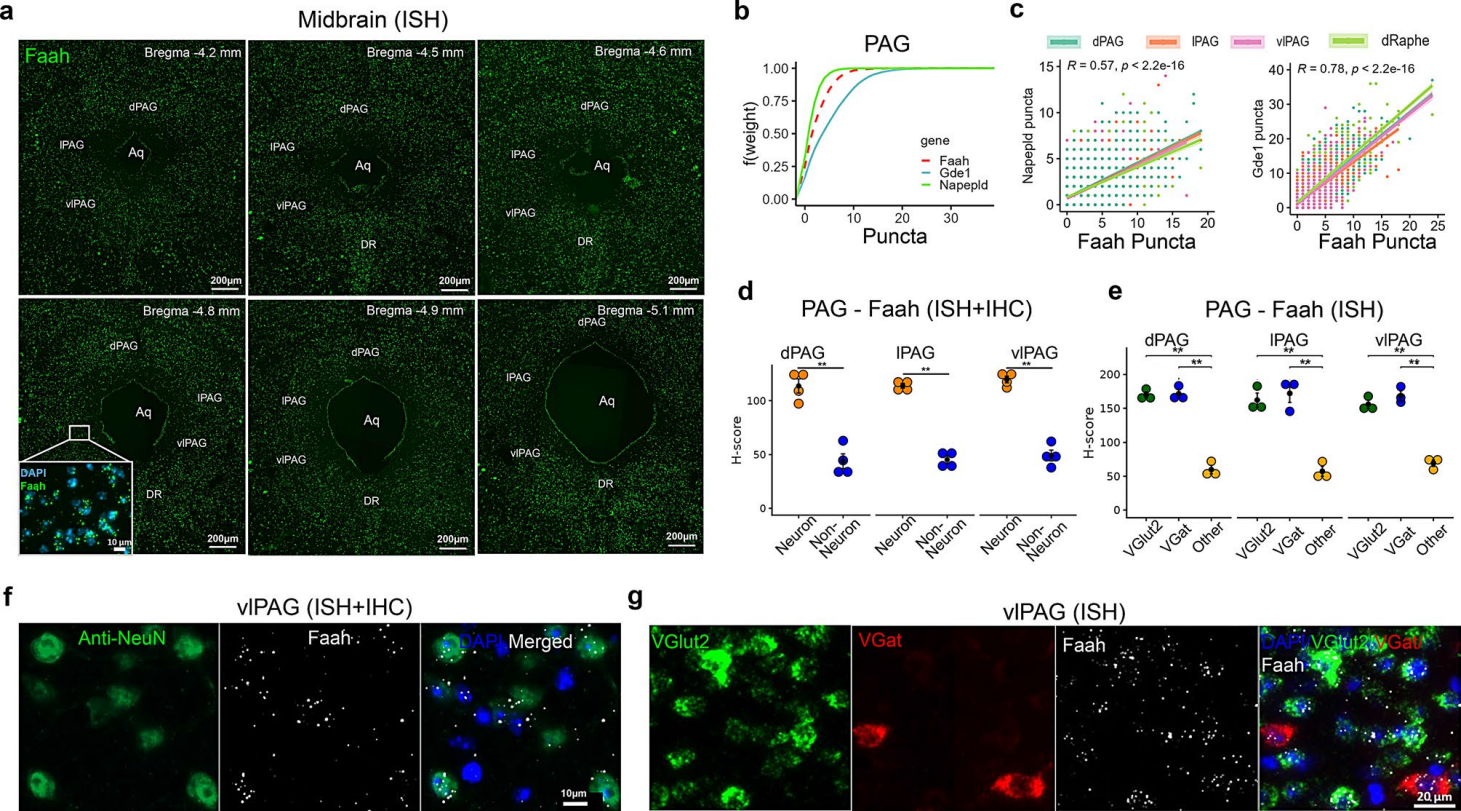
Spatial distribution of AEA metabolizing enzyme transcripts in the midbrain periaqueductal gray (PAG) and dorsal raphe (DR). (**a**) Distribution of AEA hydrolyzing enzyme, *Faah*, in the rostro-caudal extent of the PAG, with an inset representing *Faah* ISH staining in DAPI+ cells in the ventrolateral PAG (vlPAG). (**b**) empirical cumulative distribution function (ECDF) of puncta counts of AEA synthesizing and hydrolyzing enzymes in the PAG shows predominance of *Gde1* compared to *Faah* and *Napepld* (*Gde1*>*Faah*>*Napepld*). (**c**) Correlation between AEA synthesizing enzymes (*Napepld* or *Gde1*) and AEA hydrolyzing enzymes (*Faah*) puncta counts in the different subdivisions of the PAG and DR. (**d**) Comparison of *Faah* H-score (ISH) between neurons (NeuN+) and non-neuronal cells (DAPI) in the PAG. (**e**) Comparison of *Faah* H-score between *VGlut2*+, *VGat*+ neurons and other cell types in the PAG. The H-score represents a weighted histological score. (**f**) Representative images from dual ISH and IHC show presence of Faah puncta (white) in NeuN+ (green) and DAPI+ (blue) cells. (**g**) The ISH image panel shows predominant presence of Faah puncta (white) in both *Vglut2*+ (green) and *Vgat*+ (red) neurons in the ventrolateral PAG (vlPAG) compared to DAPI+ (blue) cells. Abbreviations: dPAG - dorsal PAG; lPAG - lateral PAG; vlPAG - ventrolateral PAG; aq - aqueduct. Data in (**d–e**) are presented as mean ± SEM (*n* = 3–4 mice/cell type). *p < 0.05, **p < 0.01, *** *p* < 0.001 and **** *p* < 0.0001

**Fig. 13:**
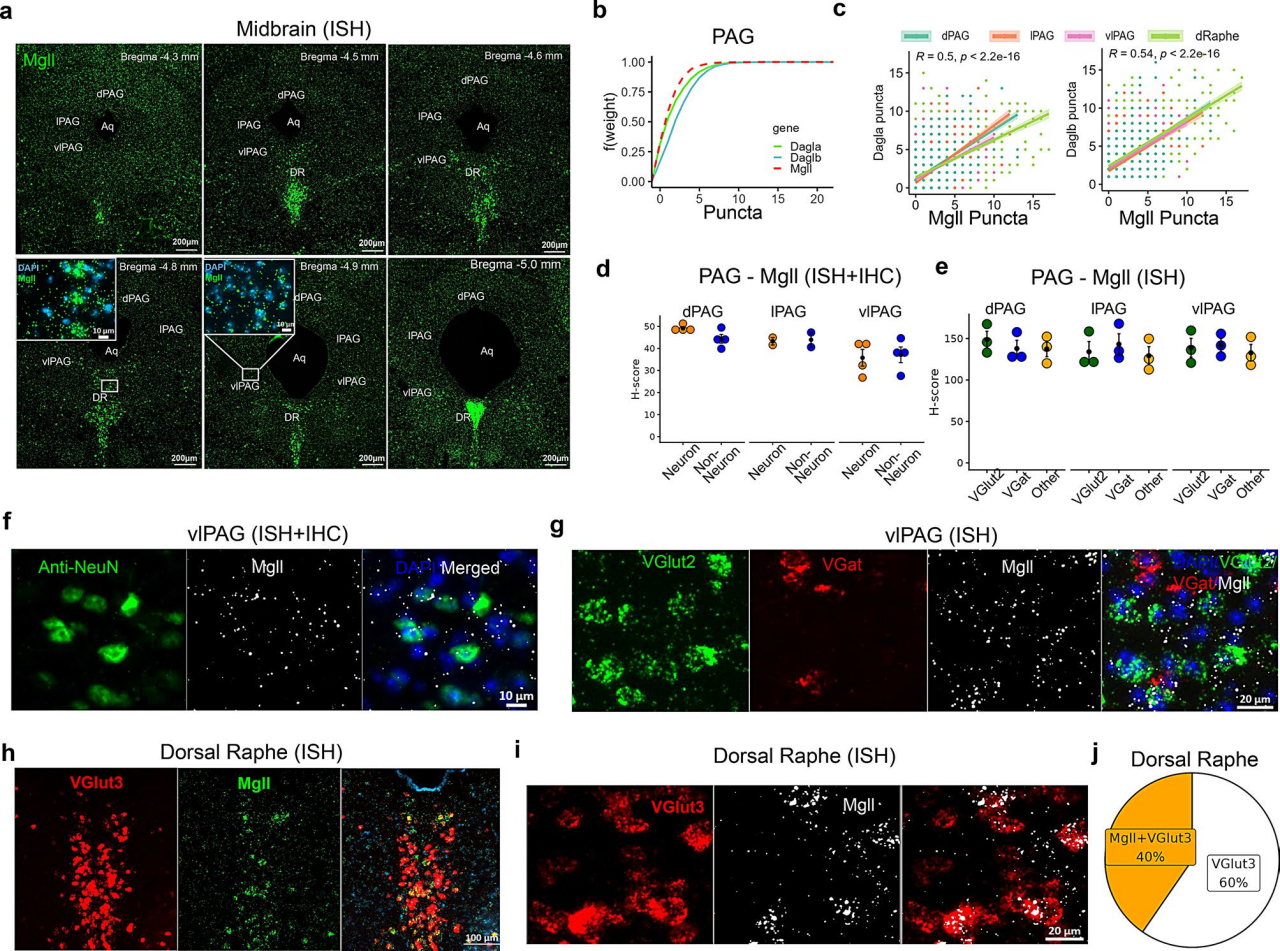
Spatial distribution of 2-AG metabolizing enzyme transcripts in the midbrain, periaqueductal gray (PAG) and dorsal raphe (DR). (**a**) ISH distribution of *Mgll* in the rostro-caudal extent of the PAG and DR, with insets showing predominance of *Mgll* in DAPI+ cells in the DR compared to the ventrolateral PAG (vlPAG). (**b**) Empirical cumulative distribution function (ECDF) of 2-AG synthesizing and hydrolyzing enzyme puncta counts in the PAG shows *Daglb* > *Dagla* > *Mgll*. (**c**) Correlation between 2-AG synthesizing enzymes (*Dagla* or *Daglb*) and 2-AG hydrolyzing enzyme (*Mgll*) puncta counts in different subdivisions of the PAG and DR. (**d**) Comparison of *Mgll* H-score present in neurons (NeuN+) and non-neuronal cells (DAPI) of the PAG. (**e**) Comparison of *Mgll *H-score among *VGlut2*+, *VGat*+ neurons and other cell types (DAPI) in the PAG. (**f**) Representative images from dual ISH and IHC show presence of *Mgll* puncta in NeuN+ and DAPI+ cells. (**g**) ISH image panel shows presence of *Mgll* puncta in both *Vglut2*+ and *Vgat*+ neurons in the ventrolateral PAG (vlPAG). (**h**, **i**) Images show ISH distribution of *Mgll* within VGlut3+ neurons in the DR. (**j**) The pie chart represents the proportion of *VGlut3*+ neurons (*n* = 2578) containing 2-AG hydrolyzing enzyme (*Mgll*) in the DR. Abbreviations: dPAG - dorsal PAG; lPAG - lateral PAG; vlPAG - ventrolateral PAG; DR - dorsal raphe; aq - aqueduct. ISH data (H-score, *n* = 3–4 mice/cell type) are presented as mean ± SEM

We analyzed gene expression levels in different subdivisions of the PAG (dorsal PAG - dPAG; lateral PAG - lPAG; ventrolateral PAG - vlPAG) and the DR (Figs. 12a and 13a). Similar to the TG and TNC, the expression of AEA metabolizing enzymes differed in the PAG, following the pattern *Gde1* > *Faah* > *Napepld* (Fig. 12b). The proportion of cells co-expressing both *Gde1*/*Faah* or *Napepld*/*Faah* was high across all PAG subdivisions. However, *Napepld* expression was slightly higher in the dPAG compared to lPAG or vlPAG, while *Gde1* expression was lower in the lPAG compared to other subdivisions (Fig. 12c). ISH and IHC analyses revealed that *Faah* transcripts were abundant in NeuN+ cells (Fig. 12d and f, *p* < 0.001), and *Faah* was equally present in the *VGlut2*+ and *VGat*+ neurons (Fig. 12e and g). Despite no significant difference in *Faah* expression between *VGlut2*+ and *VGat*+ neurons, both neuronal types expressed significantly more *Faah* puncta in their soma compared to other cell types in the PAG (Fig. 12e, *p* < 0.0001).

In contrast to the TNC, *Mgll* puncta counts in the PAG were lower compared to *Dagla* and *Daglb* (Fig. 13a–c); however, *Mgll* levels were notably higher in the DR (Figs. 13a and h–j). The rostro-caudal distribution of *Mgll* expression differed subtly across PAG subdivisions, whereas the expression varied remarkably in the DR (Fig. 13c). Both *Dagla/Mgll* and *Daglb/Mgll* co-expression levels were lower in vlPAG compared to other subdivisions of the PAG.

In contrast to the PAG, *Mgll* expression in the DR was dense, with increased puncta counts in the caudal region compared to the rostral extent of the midbrain (Fig. 13a). Interestingly, unlike *Faah* in the midbrain PAG (Fig. 12d), *Mgll* expression was comparable between neuronal and non-neuronal cells (Fig. 13d–g, *p* > 0.05). While *Mgll* was present in both *VGlut2*+ and *VGat*+ neurons in the PAG (Figs. 13e and g), within the DR, *Mgll* was mostly present in *VGlut3*+ neurons (Fig. 13h and i, Supplementary Fig. 5). Indeed, nearly all *Mgll*-positive cells in the DR were identified as *VGlut3*+ neurons, and approximately 40% of the *VGlut3*+ neurons (*n* = 2578) contained *Mgll* transcripts (Fig. 13j).

### AEA and 2-AG cognate receptors in the midbrain

**Fig. 14:**
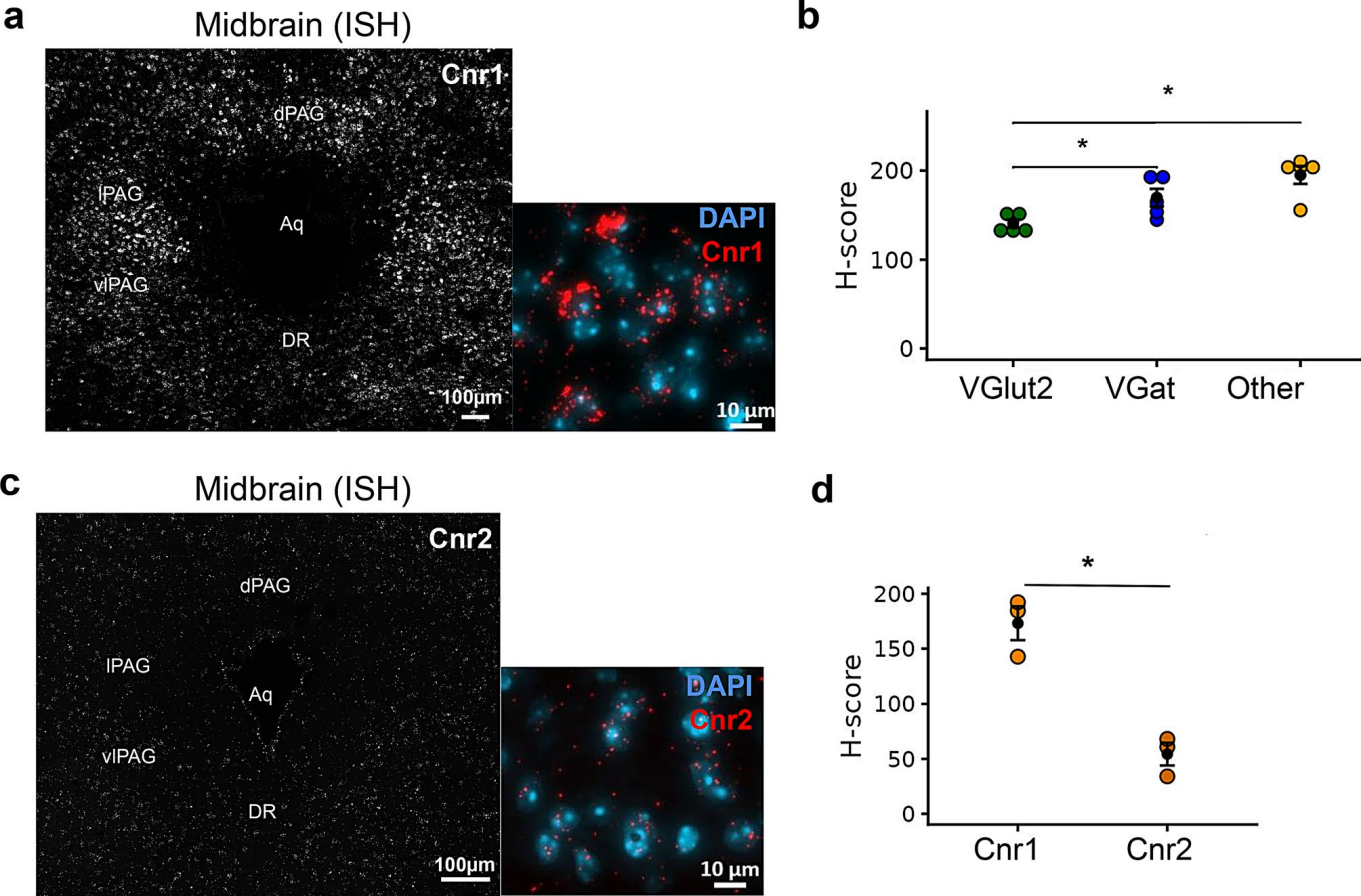
Localization of eCB cognate receptors (*Cnr1*/*Cnr2*) transcripts in the midbrain periaqueductal gray (PAG). (**a**) Distribution of *Cnr1* in all subdivisions of the PAG. (**b**) Dotplot shows comparison of ISH *Cnr1* H-score among *VGlut2*+, *VGat*+ neurons and other cell types (DAPI+ cells) in the PAG. (**c**) Image showing the sparse expression of *Cnr2* in different subdivisions of the PAG. (**d**) Comparison of H-score between *Cnr1* and *Cnr2* demonstrates the predominance of *Cnr1* in the PAG (H-score, *n* = 3–4 mice/cell type)

Expression levels of *Cnr1* were considerably higher in all subdivisions of the PAG (Fig. 14a) compared to the TNC (Fig. 7a). Particularly, consistent with earlier studies, the expression level of *Cnr1* was higher in *VGat*+ neurons than in *VGlut2*+ neurons (Fig. 14b; *p* < 0.001). Unlike in the TNC (Fig. 7e), where *Cnr1* expression was predominantly neuronal, *Cnr1* expression in the PAG was comparable to, or even higher in, non-*VGlut2*+ and non-*VGat*+ cells compared to *VGlut2*+ and *VGat*+ neurons (Fig. 14b). Similar to the TNC, sparse labeling of *Cnr2* was observed in the midbrain (Fig. 14c), where *Cnr1* expression was three-fold higher than that of *Cnr2* (Fig. 14d, *p* < 0.01; large effect size Hedges’ g = 4.18).

### TBI induced changes in the gene expression of AEA and 2-AG hydrolyzing enzymes in the midbrain

**Fig. 15:**
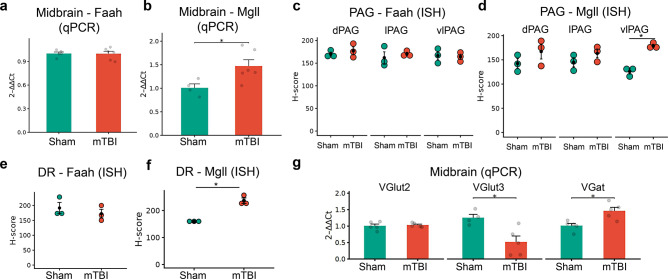
mTBI-induced changes in the gene expression of midbrain eCB hydrolyzing enzymes (Faah/mgll). a-b. Relative expression (qPCR) of eCB hydrolyzing enzymes *Faah* (**a**) and *Mgll* (**b**) 7 days following mTBI. (**c,d**) Comparison of ISH H-scores of *Faah* (**c**) and *Mgll* (**d**) between sham and mTBI groups in the different subdivisions of the PAG. (**e,f**) Comparison of eCB hydrolyzing enzymes *Faah* (**e**) and *Mgll* (**f**) ISH H-scores between sham and mTBI groups in the DR. (**g**) mTBI-induced relative gene expression (qPCR) changes in glutamate (*VGlut2*/*VGlut3*) and GABA (*VGat*) vesicular transporters in the midbrain. Relative expression data (bar graphs) are presented as mean ± SEM (*n* = 5–6 mice/group). Dot plots representing H-scores are presented as mean ± SEM (*n* = 3 mice/group). * *p* < 0.05, **p < 0.01, *** *p* < 0.001 and **** *p* < 0.0001

Region-specific upregulation of hydrolyzing enzymes was observed in the midbrain 7 days post-mTBI. No significant difference was observed in *Faah* within the PAG (qPCR - Fig. 15a; ISH - Fig. 15c) or DR (ISH - Fig. 15e). However, significant differences were observed in the *Mgll* expression in the midbrain as determined by qPCR (Fig. 15b, *p* = 0.0235) and ISH (Figs. 15d and 15f). Specifically, ISH confirmed increased *Mgll* upregulation in the DR (Fig. 15f, *p* = 0.026; large effect size Hedges’ g = 2.81) and vlPAG (Fig. 15d, p < 0.05; large effect size Hedges’ g = 5.65), and to a lesser extent in the lPAG (*p* = 0.399; Hedges’ g = 0.92) and dPAG (*p* = 0.1063; Hedges’ g = 0.881). Interestingly, this upregulation of *Mgll* appears to be a delayed response occurring at day 7, as it was not observed at day 1 post-mTBI (Supplementary Fig. 6d), a temporal profile similar to that of *Faah* (Supplementary Fig. 6a–c). Finally, we also observed mTBI-induced differential expression of vesicular glutamate (*VGlut2*/*VGlut3*) and GABA (*VGat*) transporters. Specifically, mTBI significantly downregulated *VGlut3*, but not *VGlut2*, and upregulated *VGat* gene expression in the midbrain (qPCR - Fig. 15g; p < 0.05).

## Discussion

This study characterizes classical AEA and 2-AG modulating enzymes within the trigeminal complex and midbrain. Moreover, our findings demonstrate the increased expression of specific eCB-metabolizing enzymes in the peripheral trigeminal root entry zone and in the central midbrain regions several days post-mTBI. We previously reported that mice develop allodynia/cephalic pain days after closed-head mTBI using the CHIMERA model [18, 19, 65], mirroring the late clinical onset of headache following mTBI [13]. Consistent with the progression of PTH-like symptoms, here we specifically reveal the increased expression of the endocannabinoid hydrolyzing enzymes *Faah* in peripheral TREZ, and *Mgll* in the vlPAG and DR, a week post-mTBI.

Although we showed that major metabolizing enzymes are co-expressed in the majority of cells, the predominance of specific classical and non-classical AEA and 2-AG metabolizing enzymes over others in each region underscores the complexity and the complementary pathways through which AEA and 2-AG could be metabolized [4–7]. Classically, *Napepld*, *Dagla* and *Daglb* are the primary AEA and 2-AG synthesizing enzymes, while *Faah* [78] and *Mgll* [51] are the principal AEA and 2-AG hydrolytic enzymes, respectively. Accumulating evidence indicates that Gde1, ABHD6, ABHD12, NAAA, Lysopld, protein tyrosine phosphatase non-receptor type 22 (PTPN22), Src homology 2-containing inositol phosphatase-1 (SHIP-1), Lyso-PI-PLC, and eicosanoid biosynthetic enzymes such as cyclooxygenase (COX)-2, lipoxygenases (LOXs), and cytochrome P450 enzymes are also involved in maintaining eCB tone [5]. Knockout studies have shown that 2-AG is primarily synthesized by *Dagla* in the brain and spinal cord [10, 79]. The increased abundance of *Gde1* transcripts compared to *Napepld*, and *Daglb* transcripts compared to *Dagla* in the TG, TNC and PAG, suggests that *Gde1* and *Daglb* could compensate for *Napepld* [6] and *Dagla* [79], contributing to the synthesis of AEA and 2-AG in these regions.

Since the deletion of *Gde1* and *Napepld* only partially contributes to the synthesis of AEA [6], we sought to investigate the presence of these synthesizing enzymes in the trigeminal complex. Specifically, in the TG, co-labeling of *Napepld* and *Faah* was enriched in the ophthalmic branch, whereas co-labeling of *Gde1* and *Faah* was distributed uniformly across all three TG branches. Such noticeable differences in co-labeling of synthesizing and hydrolyzing enzymes suggest that AEA may be involved in distinct, TG branch-specific functions. Particularly, sensory information from the cranial region is perceived by neurons in the ophthalmic branch of the TG [80] and relayed through the TREZ, a region classically implicated in trigeminal neuralgia [81], and headache/migraine [82]. Here, we also found that the eCB-metabolizing enzymes are present not only in the TG sensory neurons but also in the root entry zone.

Of all the major eCB-metabolizing enzymes characterized in this study, we only observed an mTBI induced increase in *Daglb* expression in the TG. Although *Daglb* is well known for its role in 2-AG synthesis, it is also involved in the production of eicosanoids that contribute to pain signaling [83], and peripherally restricted DAGL inhibitors produce anti-nociceptive effects [84]. On the other hand, in the TREZ, we observed not only increased *Faah* and *Mgll* expression but also increased gliosis 7 days after injury, which could indicate reduced eCB tone mediated by glia in mTBI mice. Since closed-head injury using CHIMERA induces cephalic pain one-week post-mTBI [18, 19], it is likely that increased eicosanoids production through DAGLb and reduced eCB tone in the TG and TREZ, respectively, play a significant role in modulating neuropathic pain following injury.

Within the brainstem, the eCB anandamide is known to modulate the activity of TG neurons and Aδ- and C-fibers in the TNC [85, 86]. Here, we have shown a modest increase in *Napepld* and *Faah* in the superficial layers compared to the deep layers of the TNC. Differences in the expression of eCB metabolizing enzymes between superficial and deep layers suggest distinct eCB modulation in the transmission of sensory information. Specifically, CB1 activation in the TNC is known to inhibit durally evoked activation of TG nociceptive Aδ- and C-fibers [86]. Although eCB may play distinct roles across the lamina of the TNC, further research is needed to fully characterize these layer specific functions. Furthermore, the predominant presence of *Faah* in the glutamatergic and GABAergic neurons compared to other cell types suggests that AEA could be modulated by these major neuronal populations. Comparably, though more strikingly, the increased presence of 2-AG synthesizing enzyme *Dagla* in the superficial layers over the deep layers suggests a profound and complex control of sensory information by the eCBs in the brainstem.

Within the TNC, although the expression of Napepld was distinct between the superficial and deep layers, we did not observe mTBI induced changes in the expression of this highly studied AEA synthesizing enzyme. However, *Gde1* was increased by 7 days post-mTBI. While it is possible that *Gde1* is involved in the synthesis of AEA, the lack of differential Faah expression following mTBI may suggest a possible alternate role for *Gde1*. Moreover, *Gde1* may play an indirect role in generating the precursors needed for lipid synthesis involved in tissue repair following mTBI [87]. On the other hand, the mTBI induced decrease in *Dagla* could imply that mTBI reduces 2-AG tone in the interneurons of the TNC [88].

Within the midbrain, we found that AEA-metabolizing enzymes were equally distributed in the PAG and DR, whereas the primary 2-AG hydrolytic enzyme was predominantly present in the DR. These regions have long been implicated in the pathogenesis of migraine and headache [80, 89, 90]. Earlier pre-clinical studies have shown that stimulation of the dorsal and lateral PAG produces analgesic effects through an increase in AEA [91] or 2-AG [50]. Interestingly, in a chronic constriction injury (CCI) model, AEA, but not 2-AG, was significantly increased in the DR [92]. Although we did not directly measure 2-AG concentrations in the PAG or DR, increased *Mgll* expression could act as a proxy for reduced 2-AG tone in the midbrain and their respective projecting regions. This can be substantiated by findings showing that enhanced *Mgll* in the midbrain correlates with reduced 2-AG levels in a migraine model [40]. This dichotomy on enhanced AEA in the peripheral model (CCI) [92] versus increased *Mgll* expression (our study) and reduced 2-AG [40] in central (mTBI) or headache models likely reflects distinct pathophysiological mechanisms depending on the origin of neuropathic pain. Clinically, less effective pain modulation in the PAG has been associated with diminished resistance to cephalic pain during migraine attacks [93]. Similarly, Delta-9-tetrahydrocannabinol (THC) has been shown to reduce chemotherapy-induced peripheral neuropathy, an effect linked to reduced connectivity between the DR and other brain regions [94]. Together, these studies suggest that eCBs in the midbrain regions are involved in modulating pain networks.

The DR is well known to modulate pain responses [95–97] and has minor projections to the PAG [98–100]. In this study, the increased presence of *Mgll* in DR *VGlut3*+ neurons may lead to impaired depolarization-induced suppression of excitation [101] or long-term potentiation of glutamatergic neurons [102]. Particularly, VGlut3 neurons in the raphe project to brain regions that modulate affective behavior [103–105]. Although DR VGlut3 neurons project to the ventral tegmental dopaminergic rewards circuit [106], a recent study showed that activation of these neurons attenuated chronic pain following spinal nerve injury [107]. Furthermore, recent studies have shown that chemogenetic activation of GABAergic neurons in the PAG leads to nociception [108, 109]. This is likely due to suppression of opioid- or cannabinoid- mediated disinhibition [110], which supports the vlPAG GABA disinhibition hypothesis [111, 112]. Accordingly, the reduced *VGlut3* expression and increased *Mgll*/*VGat* gene expression in the midbrain observed in this study appear to correlate with the enhanced cephalic pain observed in mTBI mice. Thus, the exact function of the eCB modulation through increased *Mgll* in VGlut3 neurons following mTBI warrants further investigation. Nonetheless, the descending pain pathway is well characterized [95, 113, 114], with neurons of the vlPAG playing an important role in pain modulation.

Cognate receptors of eCBs have been extensively studied throughout the peripheral and central nervous systems. Primary targets of eCBs are CB1 and CB2 receptors, whose expression levels vary throughout the brain [115, 116]; they are also present in the peripheral sensory ganglia, where receptors are synthesized and transported to the peripheral nerves [117]. The presence of CB1 receptors is well documented in different layers of the spinal dorsal horn, where they are present in neurons at the transcript level [41] and in the nerve fibers at the protein level [118]. Particularly, immunohistochemical data show that about a third of glutamatergic neurons and about 20% of GABAergic neurons express CB1 in axon terminals of the spinal dorsal horn [119]. Here, we report that in the TNC, *Cnr1* is expressed in ~25% of glutamatergic neurons and 11% of GABAergic neurons, the vast majority of which are interneurons [88]. Despite low expression levels, earlier reports have identified *Cnr2* in brainstem motor neurons [54] and microglia in different brain regions (for review see [77]). Here, we report that *Cnr2* is not exclusively present in microglia in the brainstem TNC. Although we did not find mTBI-induced changes in *Cnr1* expression in the TNC, it is well documented that changes in eCB tone are dependent on the type of injury [120]. Therefore, nociception is likely mediated by a complex interplay among interneurons, endocannabinoids, and descending pain pathways [56].

### Limitations

The current study was focused on the characterization of the gene expression of highly studied eCB metabolizing enzymes in three regions of pain pathway, as well as their changes following mTBI. However, other eCB metabolizing enzymes [5, 7], such as NAAA, ABHD6 and ABHD12, may have different expression profiles and biological functions [40, 64] and were not characterized in this study. Furthermore, the preclinical model used in this study involves only male mice. Thus, our findings are limited to male specific regional differences in eCB metabolizing gene expression in both naive mice and mTBI mice. Evidence, however, shows that PTH pathology disproportionately affects females compared to males [121, 122]. In parallel, sex difference has been reported in the midbrain regions of naïve rodents [123], where female rats exhibit variability in eCB expression during the estrous cycle. This aligns with clinical case reports noting the exacerbation of migraine during menstrual phase in mTBI patients [23]. Hence, a systematics and complex characterization of eCB metabolizing gene expression changes in females, particularly across the estrous/menstrual cycle and its relation to PTH pathology warrants further investigation. Nonetheless, the current study lays a crucial foundation regarding the region-specific expression and mTBI induced sub-acute changes in the eCB metabolizing enzymes.

## Conclusions

In summary, this study provides a comprehensive characterization of the major endocannabinoid-metabolizing enzymes and receptors within the trigeminal nociceptive system and key descending modulatory centers. We demonstrate that repetitive mTBI drives a distinct, and region-specific pathology characterized by the upregulation of eCB-hydrolyzing enzymes. Specifically, the elevation of *Faah* and *Mgll* in the trigeminal root entry zone (TREZ), coupled with a delayed, sub-acute upregulation of *Mgll* in the midbrain DR and PAG, suggests a spatiotemporal dysregulation of eCBs that coincides with maintenance of cephalic pain following mild head injury.

## Electronic supplementary material

Below is the link to the electronic supplementary material.

Supplementary Material 1

Supplementary Material 2

Supplementary Material 3

Supplementary Material 4

Supplementary Material 5

Supplementary Material 6

Supplementary Material 7

Supplementary Material 8

## Data Availability

Data is available from the corresponding author upon a reasonable request.

## Abbreviations

2-AG: 2-Arachidonoylglycerol
AEA: N-Arachidonylethanolamine
CHIMERA: Closed-head impact model of engineered rotational acceleration
Dagla: Diacylglycerol lipase a
Daglb: Diacylglycerol lipase b
dPAG: Dorsal periaqueductal gray
DR: Dorsal raphe
eCB: Endocannabinoids
ECS: Endocannabinoid system
Faah: Fatty acid amide hydrolase
Gapdh: Glyceraldehyde 3-phosphate dehydrogenase
Gde1: Glycerophosphodiester phosphodiesterase 1
Iba-1: Ionized calcium binding adaptor molecule 1
ISH: In-situ hybridization
lPAG: Lateral periaqueductal gray
Mgll: Monoacylglycerol lipase
mTBI: Mild traumatic brain injury
Napepld: N-acyl phosphatidylethanolamine phospholipase D
NeuN: Neuronal nuclei encoding *RBFOX3* gene
PAG: Periaqueductal gray
PTH: Post-traumatic headache
TG: Trigeminal ganglion
TNC: Trigeminal nucleus caudalis
TREZ: Trigeminal root entry zone
Vgat: Vesicular GABA transporter encoded by *SLC32A1* gene
VGlut2: Vesicular glutamate transporter 2 encoded by *SLC17A6* gene
VGlut3: Vesicular glutamate transporter 3 encoded by *SLC17A8* gene
vlPAG: Ventrolateral periaqueductal gray

## Declarations

### Disclaimer

The statements and data in this manuscript are solely those of the authors and do not reflect the views or policies of the Department of War, the Uniformed Services University of the Health Sciences, or the Henry M. Jackson Foundation for the Advancement of Military Medicine, Inc.
