# Supplementary figures and images for: Distribution and subacute modulation of endocannabinoid metabolizing enzymes in the trigeminal complex and midbrain in a pre-clinical model of post-traumatic headache
Source: J Headache Pain. 2026 Apr 11;27(1):113. doi: 10.1186/s10194-026-02356-5 (PMC13097742; doi:10.1186/s10194-026-02356-5)

## QuPath - Custom detection of TG sensory neurons

**a**

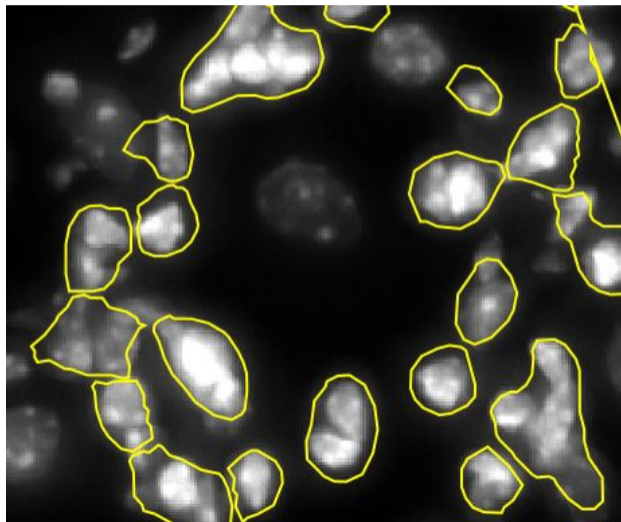

**b**

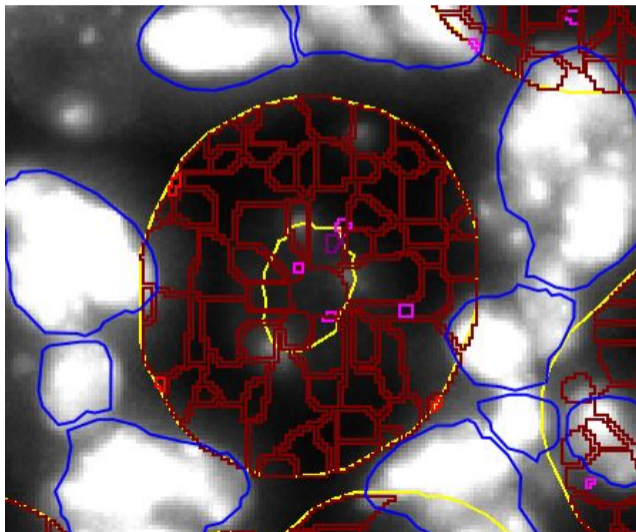

**c**

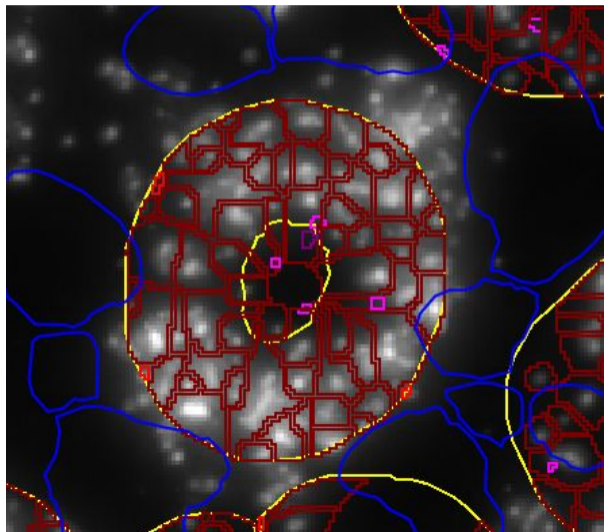

Supplement: Supplementary file 1 — Supplementary Material 1 [file 10194_2026_2356_MOESM1_ESM.pdf]

**a** TREZ (IHC)

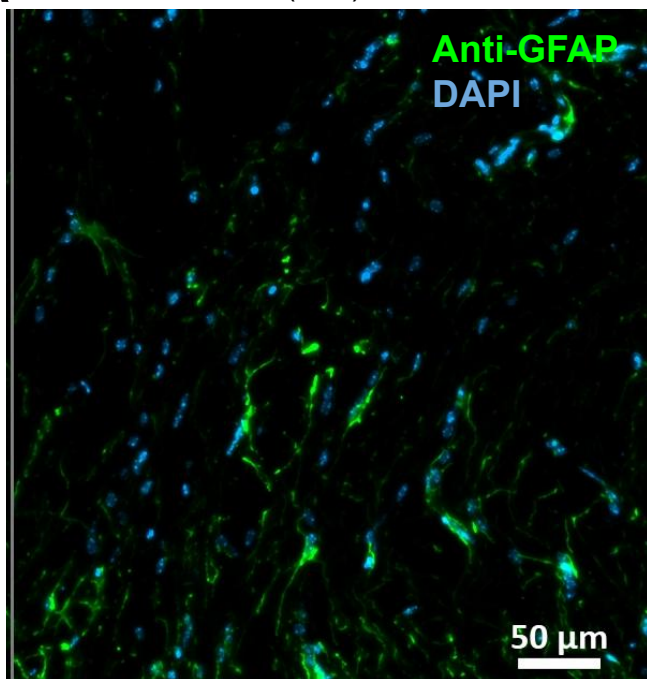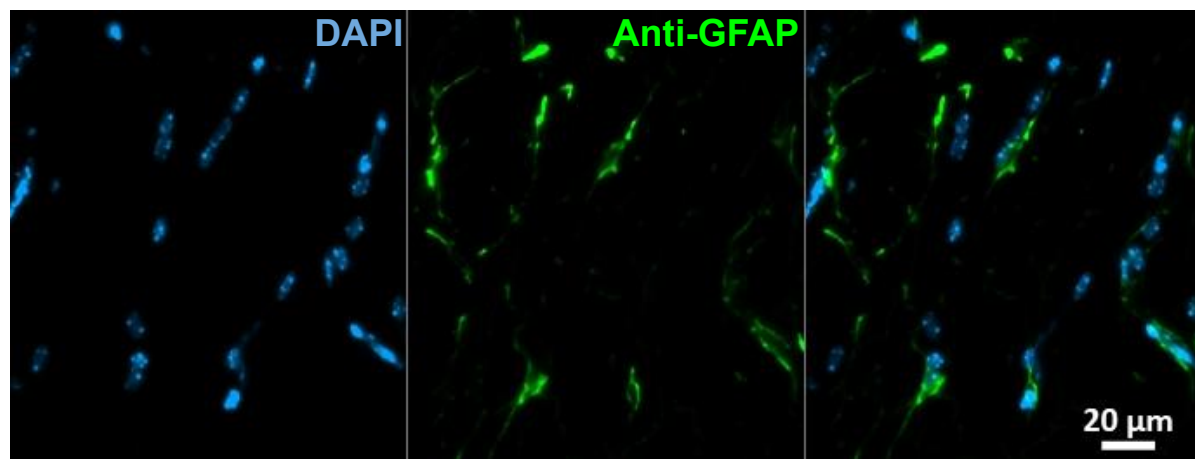

**b** TREZ (IHC)

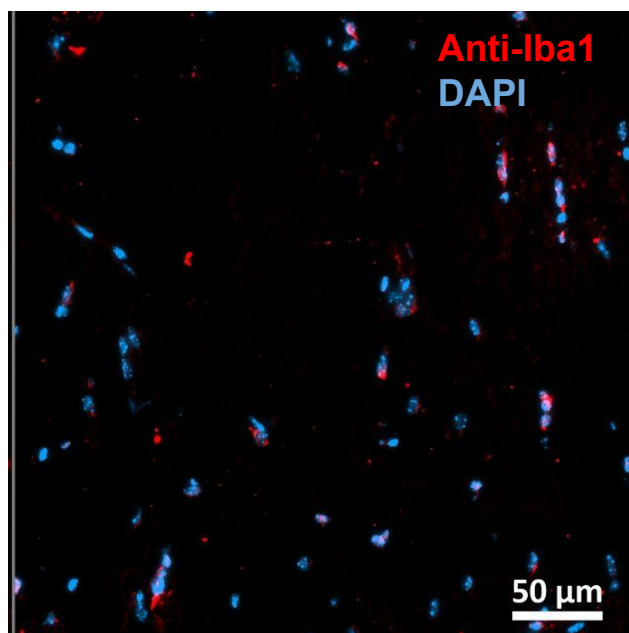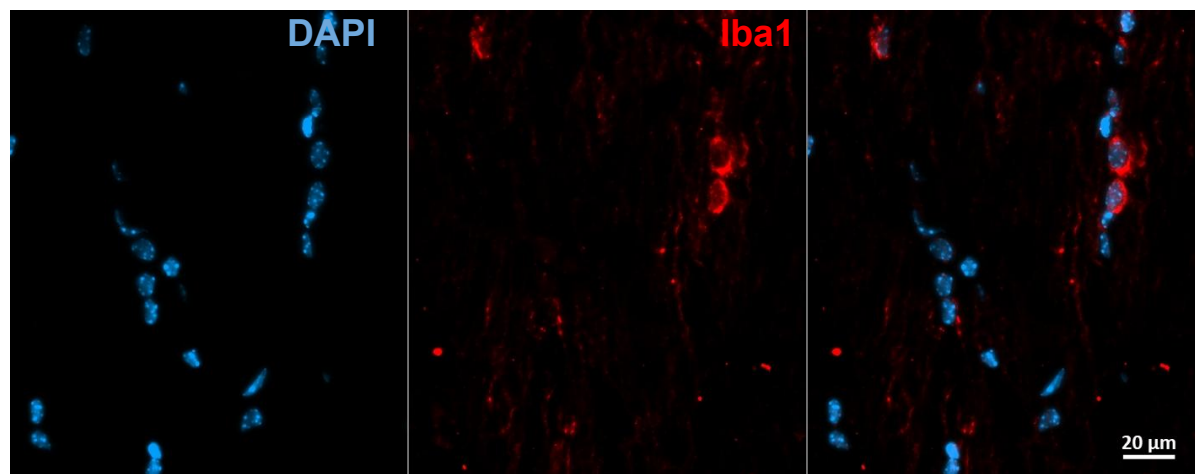

Supplement: Supplementary file 4 — Supplementary Material 4 [file 10194_2026_2356_MOESM4_ESM.pdf]

# Midbrain (ISH)

a

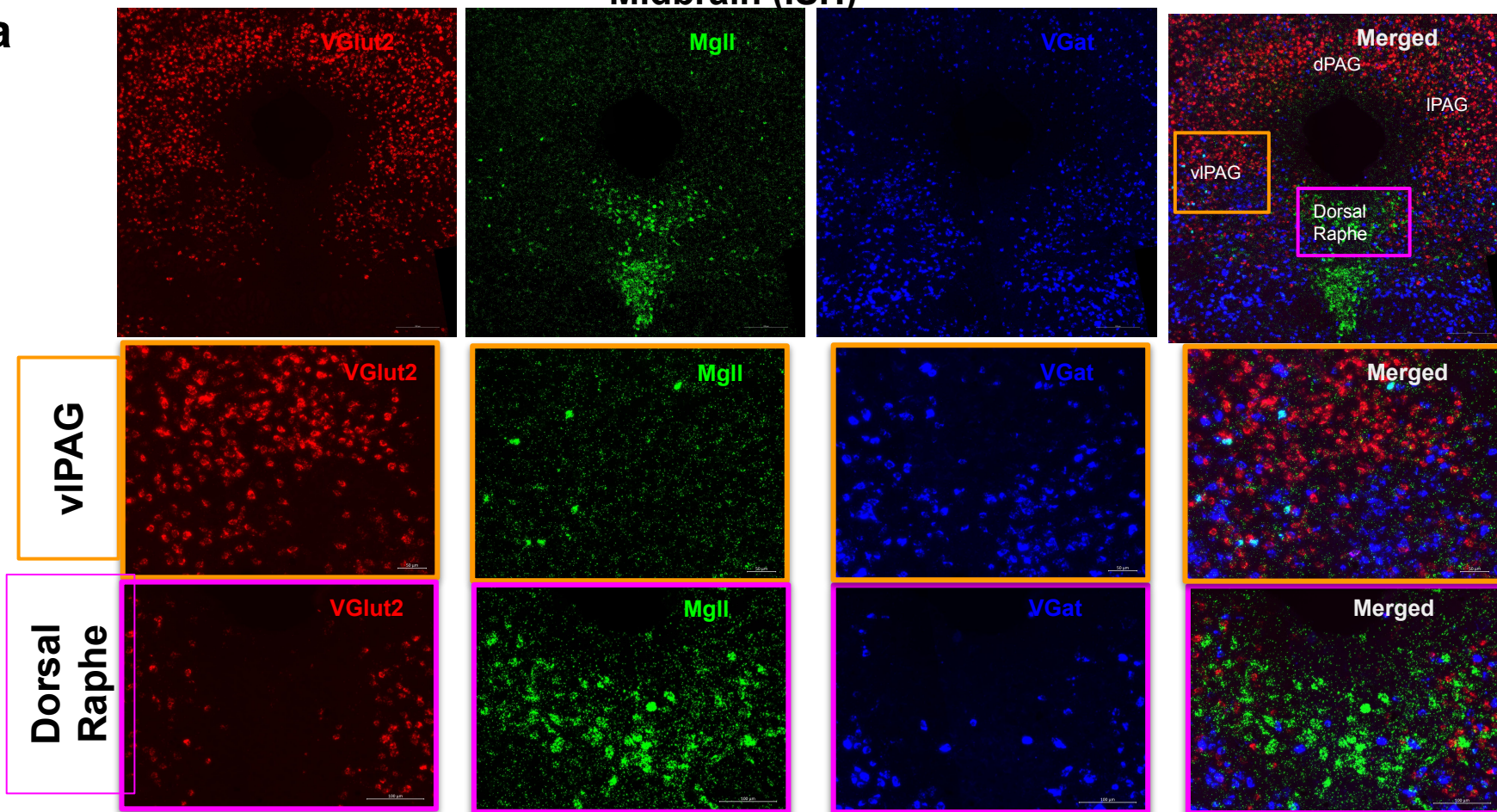

b

# Dorsal Raphe (ISH)

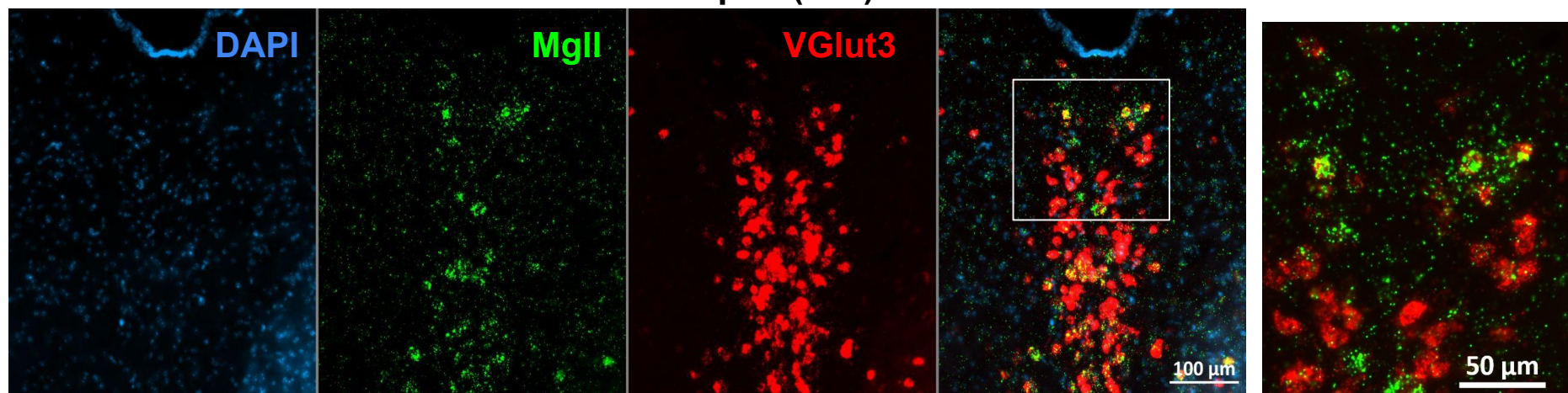

Supplement: Supplementary file 5 — Supplementary Material 5 [file 10194_2026_2356_MOESM5_ESM.pdf]

**a**

### PAG (Faah - Sham vs TBI)

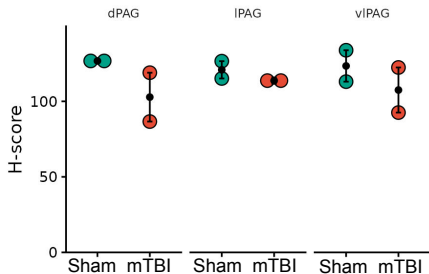**b**

### DR (Faah - Sham vs TBI)

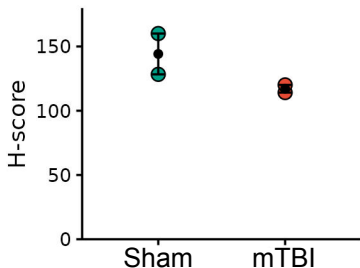**c**

### PAG (MgII - Sham vs TBI)

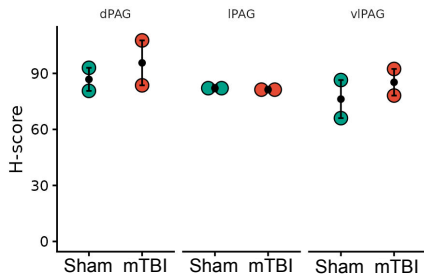**d**

### DR (MgII - Sham vs TBI)

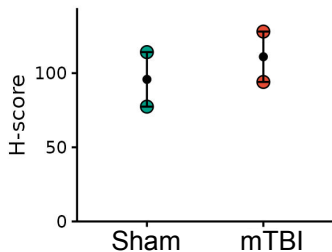

Supplement: Supplementary file 6 — Supplementary Material 6 [file 10194_2026_2356_MOESM6_ESM.pdf]
