# Supplementary material for: Distribution and subacute modulation of endocannabinoid metabolizing enzymes in the trigeminal complex and midbrain in a pre-clinical model of post-traumatic headache
Source: J Headache Pain. 2026 Apr 11;27(1):113. doi: 10.1186/s10194-026-02356-5 (PMC13097742; doi:10.1186/s10194-026-02356-5)
Supplement: Supplementary file 2 — Supplementary Material 2 [file 10194_2026_2356_MOESM2_ESM.pdf]

TG Atlas - eCB Comparison

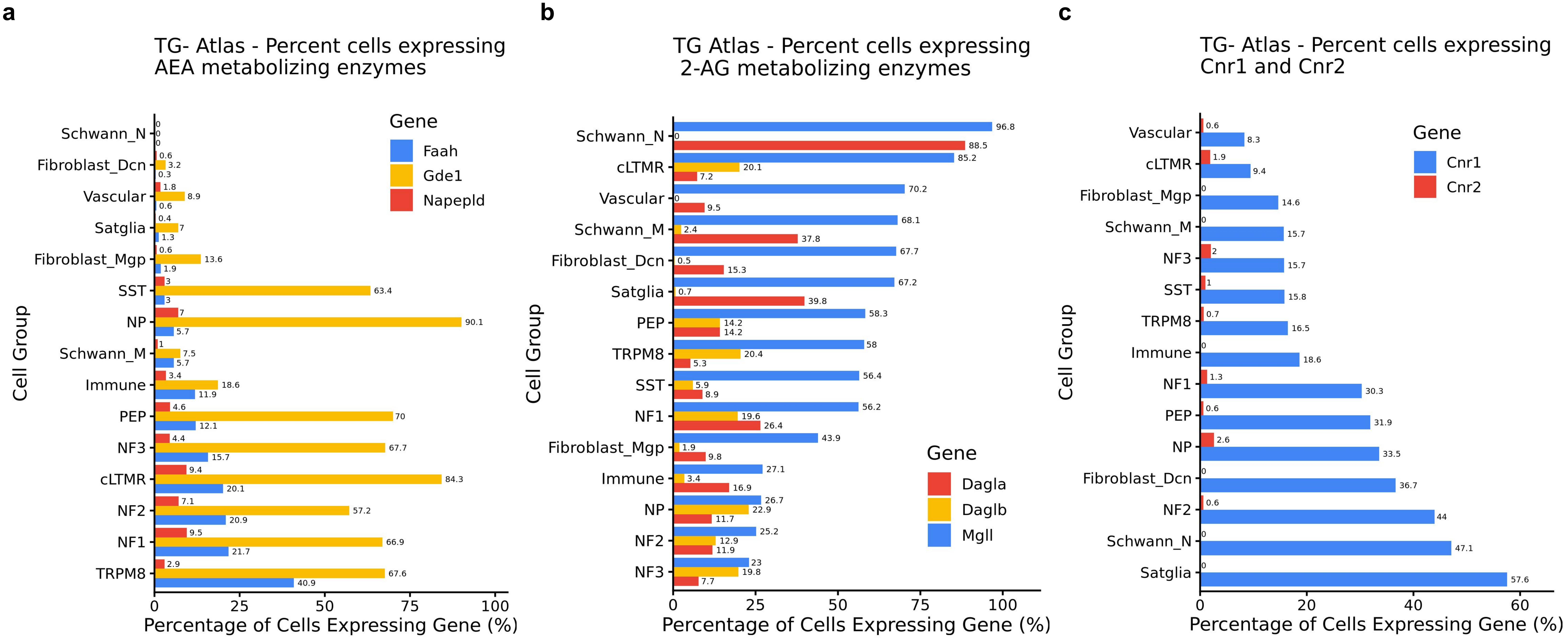

d

Cell_Group	nCells (Total)	nExpress_ Faah	nExpress_ Napepld	nExpress_ Gde1	nExpress_ Mgll	nExpress_ Dagla	nExpress_ Daglb	nExpress_ Cnr2	nExpress_ Cnr1
**cLTMR**	318	64	30	268	271	23	64	6	30
**NF1**	1987	431	189	1330	1117	525	389	26	602
**NF2**	1137	238	81	650	286	135	147	7	500
**NF3**	248	39	11	168	57	19	49	5	39
**NP**	760	43	53	685	203	89	174	20	255
**PEP**	520	63	24	364	303	74	74	3	166
**SST**	101	3	3	64	57	9	6	1	16
**TRPM8**	892	365	26	603	517	47	182	6	147
**Satglia**	1075	14	4	75	722	428	8	0	619
**Schwann_M**	1035	59	10	78	705	391	25	0	162
**Schwann_N**	157	0	0	0	152	139	0	0	74
**Fibroblast_Dcn**	619	2	4	20	419	95	3	0	227
**Fibroblast_Mgp**	478	9	3	65	210	47	9	0	70
**Immune**	59	7	2	11	16	10	2	0	11
**Vascular**	168	1	3	15	118	16	0	1	14
