# Supplementary material for: Distribution and subacute modulation of endocannabinoid metabolizing enzymes in the trigeminal complex and midbrain in a pre-clinical model of post-traumatic headache
Source: J Headache Pain. 2026 Apr 11;27(1):113. doi: 10.1186/s10194-026-02356-5 (PMC13097742; doi:10.1186/s10194-026-02356-5)
Supplement: Supplementary file 3 — Supplementary Material 3 [file 10194_2026_2356_MOESM3_ESM.pdf]

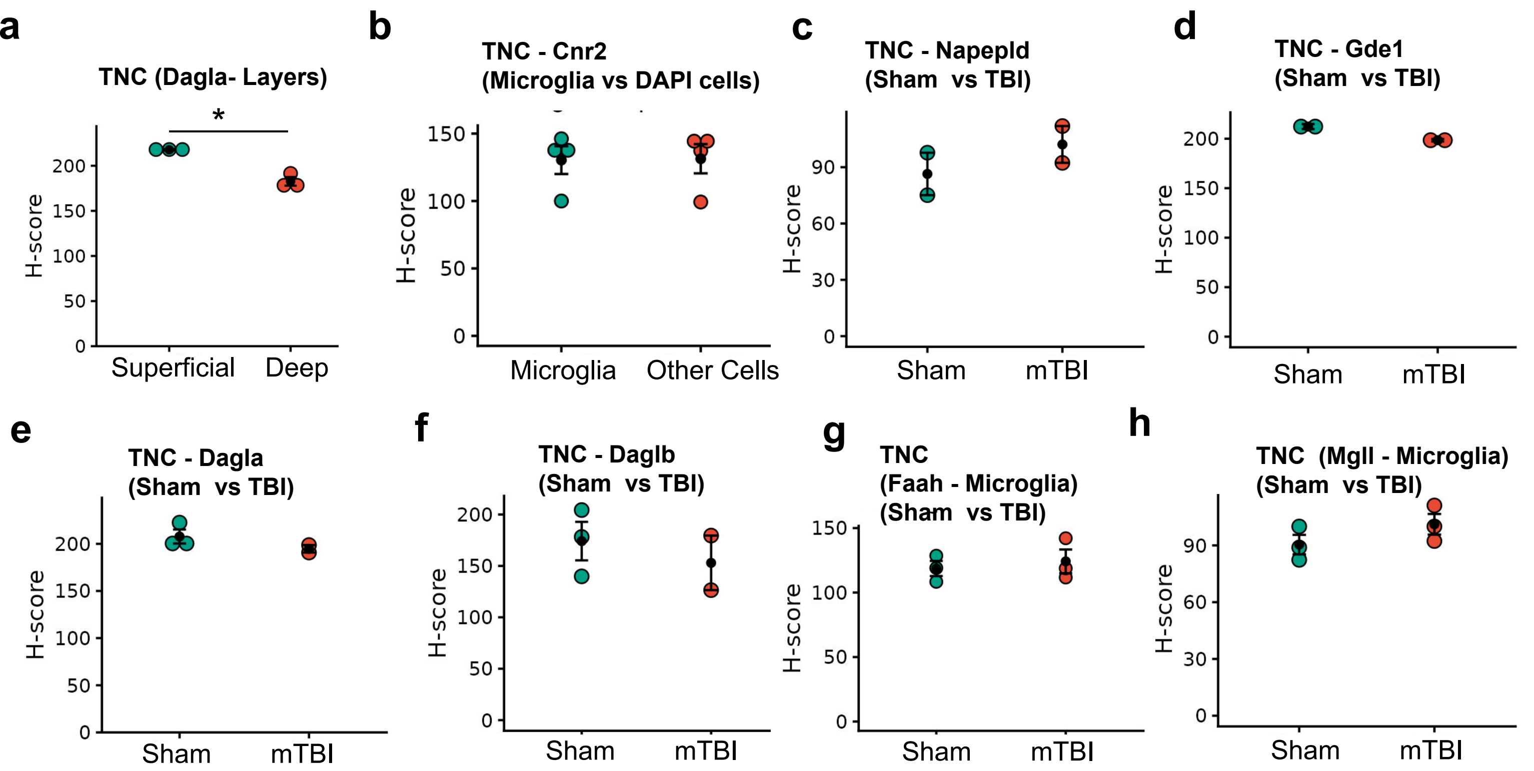
a
TNC (Dagla- Layers)
*
H-score
Superficial
Deep
b
TNC - Cnr2
(Microglia vs DAPI cells)
H-score
Microglia
Other Cells
c
TNC - Napepld
(Sham vs TBI)
H-score
Sham
mTBI
d
TNC - Gde1
(Sham vs TBI)
H-score
Sham
mTBI
e
TNC - Dagla
(Sham vs TBI)
H-score
Sham
mTBI
f
TNC - Daglb
(Sham vs TBI)
H-score
Sham
mTBI
g
TNC
(Faah - Microglia)
(Sham vs TBI)
H-score
Sham
mTBI
h
TNC (Mgll - Microglia)
(Sham vs TBI)
H-score
Sham
mTBI
