# Supplementary material for: Distribution and subacute modulation of endocannabinoid metabolizing enzymes in the trigeminal complex and midbrain in a pre-clinical model of post-traumatic headache
Source: J Headache Pain. 2026 Apr 11;27(1):113. doi: 10.1186/s10194-026-02356-5 (PMC13097742; doi:10.1186/s10194-026-02356-5)
Supplement: Supplementary file 7 — Supplementary Material 7 [file 10194_2026_2356_MOESM7_ESM.pdf]

Supplementary Table 1

List of gene probes used in RNAscope *in-situ* hybridization assays

Gene	Cat No	Accession No.
Napepld	551691-C3	NM_001359964.1
Gde1	1754271-C3	NM_019580.4
Faah	453391-C2	NM_010173.4
Dagla	478821-C3	NM_198114.2
Daglb	497801-C4	NM_144915.3
Mgll	478831-C2	NM_001166251.1
Cnr1	457341-C4	NM_007726
Cnr2	407351-C2	NM_009924.3
Slc17a6/*VGlut2*	319171-C3	NM_080853.3
Slc17a8/*VGlut3*	431261	NM_182959.3
Slc32a1/*VGat*	319191-C4	NM_009508.2
