# Supplementary material for: Distribution and subacute modulation of endocannabinoid metabolizing enzymes in the trigeminal complex and midbrain in a pre-clinical model of post-traumatic headache
Source: J Headache Pain. 2026 Apr 11;27(1):113. doi: 10.1186/s10194-026-02356-5 (PMC13097742; doi:10.1186/s10194-026-02356-5)
Supplement: Supplementary file 8 — Supplementary Material 8 [file 10194_2026_2356_MOESM8_ESM.pdf]

Supplementary Table 2

List of primes used in quantitative PCR

Genes	Forward primer	Reverse Primer
Gapdh	AGGTCGGTGTGAACGGATTTG	TGTAGACCATGTAGTTGAGGTCA
Napepld	AGCGCCAAGCTATCAGTATCC	ACGTCCTCCTCTAGTCTGTAATC
Gde1	AATGGAGCAACAGGTGTGGAG	AAATGTCAGATCACATAGTCGGC
Faah	CAACTACACCATGCCCACTC	GACCTCCAGGGCATAAGGTA
Dagla	TCCTGTCTGTGGTGCTCTTC	CCATTTCGGCAATCATACAG
Daglb	ACTCAGATTTGCCTGCCTCT	GCCTACAAGTCCCAACACCT
Mgll	AGTGCTCTGCTCACATCCTG	AGTGACAAACCAGTGACCCA
VGlut1/*Slc17a7*	AGGCTCGCCTAACCAATTCT	TTTCCCTCAGAAACGCTGGT
VGlut2/*Slc17a6*	ACGTAGGTACATAGAGGAGAGCA	AATTATCGCGTAGACGGGCA
VGlut3/*SLC17A8*	GTAAGGTGGGTCTCTTGTCAGC	ACAGCGGTTGTGGTCAAAATCT
VGat/*Slc32a1*	GCAGAAGTCCTGGTCCATCA	TTGGACACGGCCTTGAGATT
